# A Meta-Epidemiological Appraisal of the Effects of Interdisciplinary Multimodal Pain Therapy Dosing for Chronic Low Back Pain

**DOI:** 10.3390/jcm8060871

**Published:** 2019-06-18

**Authors:** Elena Dragioti, Mathilda Björk, Britt Larsson, Björn Gerdle

**Affiliations:** 1 Pain and Rehabilitation Centre, and Department of Medical and Health Sciences, Linköping University, SE-581 85 Linköping, Sweden; mathilda.bjork@liu.se (M.B.); britt.larsson@liu.se (B.L.); bjorn.gerdle@liu.se (B.G.); 2 Division of Occupational Therapy, Department of Social and Welfare Studies, Faculty of Health Sciences, Campus Norrkoping, Linköping University, SE-60174 Linköping, Sweden

**Keywords:** programme dosage, interdisciplinary multimodal pain therapy, pain rehabilitation, low back pain, meta-analysis

## Abstract

Using a meta-analysis, meta-regression, and a meta-epidemiological approach, we conducted a systematic review to examine the influence of interdisciplinary multimodal pain therapy (IMPT) dosage on pain, disability, return to work, quality of life, depression, and anxiety in published randomised controlled trials (RCTs) in patients with non-specific chronic low back pain (CLBP). We considered all RCTs of IMPT from a Cochrane review and searched PubMed for additional RCTs through 30 September 2018. A subgroup random-effects meta-analysis by length, contact, and intensity of treatment was performed followed by a meta-regression analysis. Using random and fixed-effect models and a summary relative odds ratio (ROR), we compared the effect sizes (ES) from short-length, non-daily contact, and low-intensity RCTs with long-length, daily contact, and high-intensity RCTs. Heterogeneity was quantified with the I^2^ metric. A total of 47 RCTs were selected. Subgroup meta-analysis showed that there were larger ES for pain and disability in RCTs with long-length, non-daily contact, and low intensity of treatment. Larger ES were also observed for quality of life in RCTs with short-length, non-daily contact, and low intensity treatment. However, these findings were not confirmed by the meta-regression analysis. Likewise, the summary RORs were not significant, indicating that the length, contact, and intensity of treatment did not have an overall effect on the investigated outcomes. For the outcomes investigated here, IMPT dosage is not generally associated with better ES, and an optimal dosage was not determined.

## 1. Introduction

Currently, interdisciplinary multimodal pain therapy (IMPT) is used as a first-line therapy for chronic low back pain (CLBP) management [1,2,3,4]. IMPT, a long biopsychosocial treatment framework provided by a team of professionals, generally contains a synchronised combination of physical, educational, or psychological treatments in combination with measures for returning to work/studies [5,6]. Because IMPT can effectively treat patients with non-specific CLBP, it is strongly recommended [1,2,3,4]. Compared to usual care or physical treatment, IMPT has a consistent positive effect on disability and pain according to systematic reviews (SRs) [2,4]. In a new umbrella review, our team found suggestive evidence that IMPT might improve the likelihood of returning to work [5]. In addition, IMPT might decrease the personal and economic burden and increase the patients’ treatment participation [2,7].

However, IMPT treatments are rather costly. The costs mainly depend on the treatment dosage, which includes the total duration, the contact (daily or non-daily), and intensity of treatment (number of contact hours per week) [8,9,10]. In addition, IMPT costs increase as the duration, contact, and intensity of treatment increase [8,9,10], and IMPT heavily depends on the involved professions. Typically, IMPT includes costs for physical therapists, psychologists, occupational therapists, physicians, and administration personnel [11]. Additionally, concerns include the costs of attending such treatments and the large societal costs of those patients who do not complete the treatments and do not return to work [11]. Therefore, the dosage of the treatment and the multidisciplinary nature of IMPT provide relatively high direct costs for both patients and the healthcare system [11]. Hence, variances in the IMPT dosage may lead to differences in both effectiveness and costs [8,9,10]. 

Presently, the optimal IMPT dosage and which dosage is efficacious for the patients with non-specific CLBP is unidentified [9], despite the need for a standardisation of such treatments [5,12]. A recent systematic review showed that IMPT dosage was never studied as a primary outcome, and its optimum dosage is currently unknown [10]. Our umbrella review also showed that a short duration of IMPT for CLBP patients with short-term and medium-term pain had the largest evidence of returning to work (highly suggestive evidence and suggestive evidence, respectively) [5]. A better understanding of how IMPT dosage is associated with outcome effects (e.g., pain, disability, and work status) should be considered when determining dosage. In turn, this could lead to better and more efficient patient care, which will benefit patients, rehabilitation facilities, insurers, and employers [8,11].

Here, we conducted a systematic review with meta-analysis and a meta-epidemiological appraisal to examine whether IMPT dosage is associated with better outcome effects in patients with non-specific CLBP.

## 2. Materials and Methods

This study was designed based on the Preferred Reporting Items for Systematic Reviews and Meta-Analyses (PRISMA) guidelines [13,14]. Because this meta-research project did not require patients to be directly involved, ethical approval was not required.

### 2.1. Literature Search and Study Selection

This study includes all randomised controlled trials (RCTs) included in Kamper et al.’s Cochrane systematic review [2]. We choose this review because it is the most recent and comprehensive review with the largest number of included RCTs. We also searched PubMed through 30 September 2018 for additional fully published RCTs in peer-reviewed journals investigating the effectiveness of IMPT on non-specific CLBP. The basic search strategy included the following key terms: chronic low back pain, interdisciplinary, multimodal pain therapy, multidisciplinary biopsychosocial rehabilitation, and randomised controlled trials (for details, see Box S1, Supplementary Materials).

We included only RCTs that (1) examined any IMPT versus any control (e.g., treatment as usual, and waiting list) or other treatment (e.g., physiotherapy and surgery), (2) included only adult men and women, (3) identified a diagnosis of a non-specific CLBP lasting more than three months, and (4) published in English expect for those included in the Kamper et al.’s review [2] as the provided data in this study were already translated into English. 

We excluded studies if they (1) used a study design other than RCT, (2) compared different IMPTs with each other (i.e., head-to-head comparisons), (3) included participants with fewer than 50% diagnosed with non-specific CLBP, (4) included a diagnosis of LBP due to cancer, infection, inflammatory arthropathy, osteoporosis, high-velocity trauma, fracture, pregnancy, rheumatoid arthritis, or rheumatic pain, and (5) provided insufficient or inadequate data for quantitative synthesis.

### 2.2. Data Extraction

One investigator (E.D.) screened titles and abstracts, assessed the eligibility, extracted data, and rated the quality of the included RCTs. These were also checked by a second author (M.B.). Any disagreements were resolved by consensus, or a third reviewer (B.G.) was consulted if disagreements persisted. The six primary outcomes of interest for this study were pain, disability, work status (return to work), quality of life, depression, and anxiety as reported by the original authors of the RCTs. We chose these outcomes because these were the most common outcomes in RCTs with adequate data for analysis. Other outcomes (e.g., fear avoidance and coping strategies) provided limited data for synthesis. Because of the same limitation (i.e., limited data for adequate synthesis), we also focused only on short-term outcomes (i.e., up to three months). The IMPT dosage was defined according to total duration (in weeks), daily contact or non-daily contact, and intensity of treatment (number of contact hours per week) [2,10].

### 2.3. Assessment of Bias

We used the updated Cochrane Back Review Group criteria [15] to rate the quality of the included RCTs, which include 12 criteria and assess the risk of bias (RoB). For each criterion, the quality of each RCT was classified as high (i.e., low RoB = 1), moderate (i.e., unclear RoB = 2), and low (i.e., high RoB = 3). Next, we evaluated an overall “risk of bias” assessment for each RCT by giving one point to each criterion when low RoB was indicated. Thus, RCTs satisfying at least six of the 12 criteria and having no having no serious flaws (e.g., 80% drop-out rate in one group) were considered as “low” risk of bias [15]. RCTs with serious flaws, or those in which fewer than six of the criteria are met were considered as having a “high” risk of bias [15]. It is important to note that the blinding is quite problematic in this field [2,10].

### 2.4. Data Synthesis and Analysis

We analysed data descriptively and conducted subgroup meta-analysis of all the outcomes of interest as listed above. Specifically, to explore the effects of IMPT dosage—i.e., the total duration (in weeks), the contact, and intensity of treatment (number of contact hours per week)—a series of random-effects meta-analyses [16] were conducted by clustering the RCTs according to the following variables: short length (in weeks; <5 weeks) vs. long length (≥5 weeks); non-daily contact vs. daily contact; and low intensity vs. high intensity (e.g., less than 30 h per week vs. more 30 h per week). Dichotomous outcomes were analysed by calculating the pooled odds ratio (OR), and continuous outcomes were analysed by calculating the standardised mean difference (SMD). Between-study heterogeneity was evaluated by Cochran’s Q test [16] and quantified with the I^2^ metric of inconsistency (low, moderate, large, and very large for values of <25, 25–49, 50–74, and >75%, respectively) [17,18]. We also calculated the 95% confidence intervals for the I^2^. We used the regression asymmetry test and funnel plots to estimate publication bias for all outcomes of interest [19]. We also performed a random-effects meta-regression analysis [20] to examine the potential moderator effect of dosage aspects, mean age of the participants, gender (female), type of control (i.e., physical activity), and RoB assessment (i.e., RCTs with low risk as previously described) on treatment effects. The dosage aspects (duration and intensity) were included in the meta-regression analysis as continuous variables for a more accurate estimation [20]. The only exception was for contact since it was only in binary form.

To further systemically assess the potential influence of IMPT dosage on the outcomes of interest, we used a meta-epidemiological approach [21,22,23] comparing the magnitude of the effect size (ES) by the treatment dosage in terms of the total duration (in weeks), the contact, and intensity of treatment (number of contact hours per week) for each outcome. To match the outcome data and allow for the synthesis of the evidence, we transformed the SMD to a logOR for the continuous outcomes [22] based on a standardised formula [24]. 

From each outcome, we calculated a summary OR for the short-duration RCTs and long-duration RCTs, for the non-daily contact and daily contact, and for the low-intensity vs. high-intensity RCTs within the eligible RCTs using fixed-effect models [22,25]. This method is more proper for examining study design factors on treatment effects [21,22,23]. All comparisons were coined so that the experimental arm was always an IMPT vs. a control arm. Next, we obtained a relative OR (ROR) within all comparisons for each outcome [22]. A ROR that exceeds 1 equates to assessments providing a more favourable response to the experimental IMPT supported by an RCT with long duration, daily contact, and high intensity compared to RCTs with short duration, non-daily contact, and low intensity. To obtain a summary ROR (sROR) across all outcomes, we combined the natural logarithm estimates of the RORs for all comparisons [21] using fixed- and random-effects models [16,25].

The statistical analyses were made using STATA version 10.0 (STATA Corp, College Station, Texas, USA); a value of *p* < 0.05 (two-tailed) was set as the level of significance.

## 3. Results

We identified 41 RCTs from Kamper et al.’s study [2]. The electronic search yielded a total of 3799 potentially eligible titles. Following the search and screening and retrieval of 126 full text articles, six additional RCTs were determined to be eligible (Figure S1, Supplementary Materials). These articles were added to the 41 RCTs included in Kamper et al. [2] to make a total of 47 included RCTs (see Supplementary Materials for the list of refences of the included studies). The two independent investigators reached a very high level of agreement (43/47 RCTs). In the whole process, from screening to data extraction, any disagreement was discussed with a third researcher (B.G.) until a consensus was reached.

### 3.1. Characteristics of Included Studies

Table 1 presents the characteristics of studies. All the 47 included studies were RCTs published from 1990 to 2017. Most studies were conducted in Europe (*n* = 34; 74%). The number of participants ranged from 20 to 542 with a median number of 134 participants per study (interquartile range (IQR = 84–195), a median age of 44 years old (IQR = 41–47), a total treatment duration ranging from 1–16 weeks with a median of five weeks (IQR = 3–7), and a number of contact hours (per week) ranging from 1–100 h (median = 10; IQR = 3–30). Most RCTs provided a non-daily contact (*n* = 26; 56%). Although the definition of CLBP varied among studies, most studies defined CLBP as back pain lasting more than three months (Table 1). The number of datasets included in the meta-analysis per outcome varied from two (anxiety) to 28 (pain).

### 3.2. Risk of Bias in Included Studies

**Figure 1:**
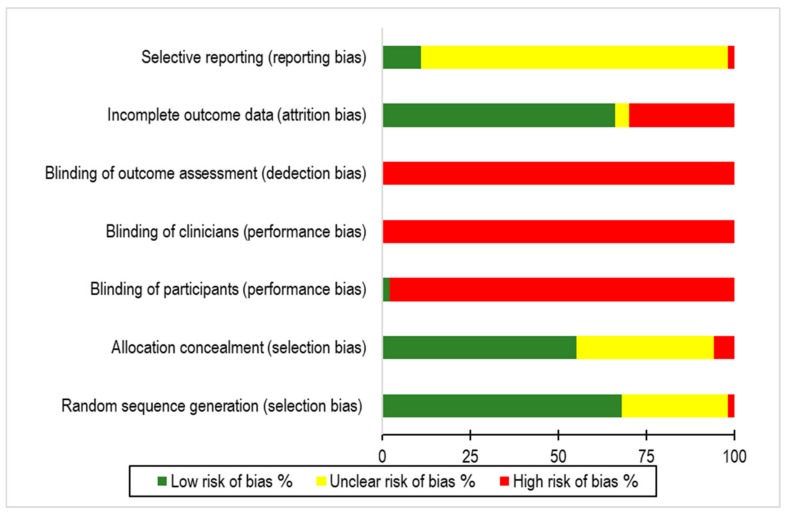
Risk of bias graph: assessments for seven risk of bias criteria presented as percentages across all included studies.

The results of the RoB assessment are presented in Table S1 (Supplementary Materials). Overall, 15 of the 47 studies (32%) were evaluated as low risk of bias. The most important methodological flaws were related to a lack of participant, clinician, and outcome assessment blinding; in particular, 46 out of 47 RCTs (almost 100%) had a high risk of bias in all three of these criteria (Figure 1). However, as in any psychotherapy [22], blinding is not possible, at least to participants or clinicians, in this type of treatment [2,10].

### 3.3. Publication Bias

Publication bias was observed for three outcomes (disability (*p* = 0.018), quality of life (*p* = 0.060), and depression (*p* = 0.081)), based on the funnel plots and Egger’s regression test (see Figures S2–S6, Supplementary Materials). No publication bias remained for the outcome of pain (*p* = 0.219) after excluding the study of Monticone et al. (2013) (Reference 27 in Supplementary Materials) providing a large outlier as seen in Figure S2 (Supplementary Materials). Publication bias for anxiety outcome could not be estimated due to the inadequate number of included studies (only two RCTs).

### 3.4. Analyses for Outcomes of Interdisciplinary Multimodal Pain Therapy (IMPT) by Length, Contact, and Intensity

For each outcome, subgroup analysis was conducted for IMPT dosage by length, contact, and intensity (Table 2). There were larger ES for pain and disability in RCTs with long length, non-daily contact, and low intensity of treatment. Larger and significant ES were also observed for quality of life in RCTs with short length, non-daily contact, and low intensity of treatment. After excluding the study of Moticone et al. (2013/2014) (References 27,28 in Supplementary Materials), the ES were similar between the aspects of dosage for the outcomes of pain and disability (see Figures S7–S12, Supplementary Materials). The forest plots of the overall ES from all studies included for the six outcomes per RoB assessment, type of control, and aspects of dosage are provided in Figures S13–S18 (Supplementary Materials).

### 3.5. Meta-Regression

In the meta-regression analyses, none of the examined variables displayed a moderating effect on the five examined outcomes (i.e., pain, disability, return to work, quality of life, and depression). For the anxiety outcome, there were insufficient observations to perform such analysis (Table S2, Supplementary Materials).

### 3.6. Comparison of Relative Odds Ratios 

**Figure 2:**
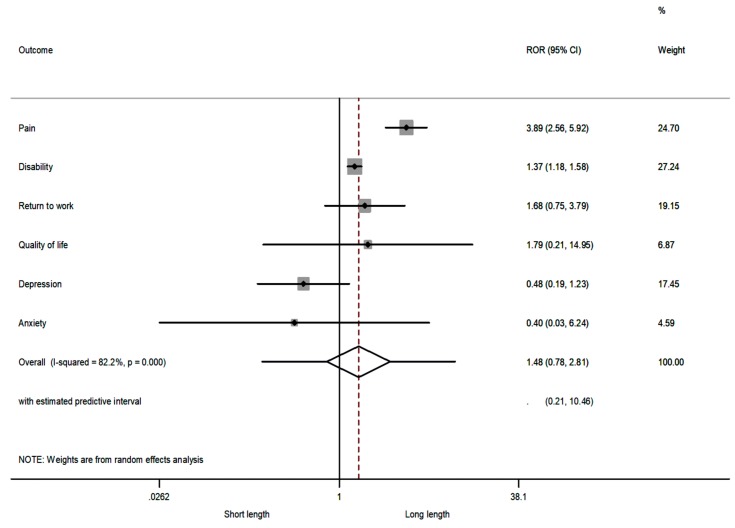
The relative odds ratios (RORs) and 95% confidence intervals (CIs) for each outcome, and the summary RORs and their 95% CIs at short term of a short-length treatment vs. long-length treatment. The RORs were calculated with a random-effects model. A ROR >1 favours long length; an ROR <1 favours short length.

The comparison of RORs by length of treatment showed that, for pain and disability, the summary RORs were >1, demonstrating that the IMPT was more favourable in RCTs with long length of treatment (i.e., RCTs with duration of more than five weeks). However, the summary ROR was not significant (sROR = 1.48 (95% confidence interval (CI) 0.78–2.81, *p* = 0.232) using the random-effects model, showing that the length of treatment did not have an overall effect on the investigated outcomes (Figure 2). Very large heterogeneity was observed (I^2^ = 82%, 95% CI 55–90%). Under the fixed-effect models the sROR was, however, significant (Figure S19, Supplementary Materials).

**Figure 3:**
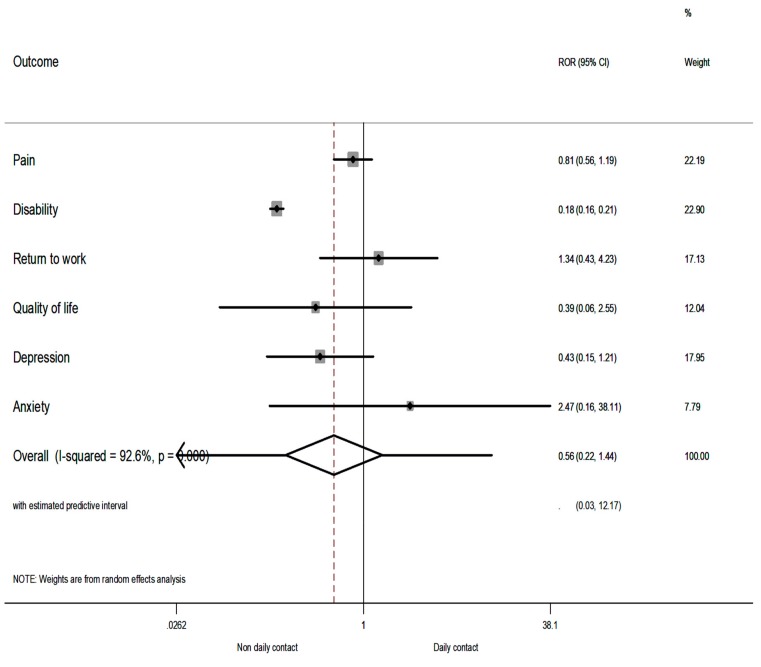
The relative odds ratios (RORs) and 95% confidence intervals (CIs) for each outcome, and the summary RORs and their 95% CIs at short term of non-daily contact vs. daily contact. The RORs were calculated with a random-effects model. A ROR >1 favours daily contact; an ROR <1 favours non-daily contact.

The comparison of RORs by contact of treatment (i.e., non-daily contact vs. daily contact) showed that the summary ROR was <1 only for disability, a finding that indicates that the IMPT was more favourable in RCTs with non-daily contact of treatment (i.e., RCTs with at least 3 h per week). However, the summary ROR was not significant (sROR = 0.56; 95% CI 0.22–1.44; *p* = 0.230) according to the random-effects model (Figure 3), showing that the contact of treatment did not have an overall effect on the investigated outcomes, whereas the sROR was significant under the fixed-effect models (Figure S20, Supplementary Materials). Very large heterogeneity was also present (I^2^ = 93%, 95% CI 88–95%).

**Figure 4:**
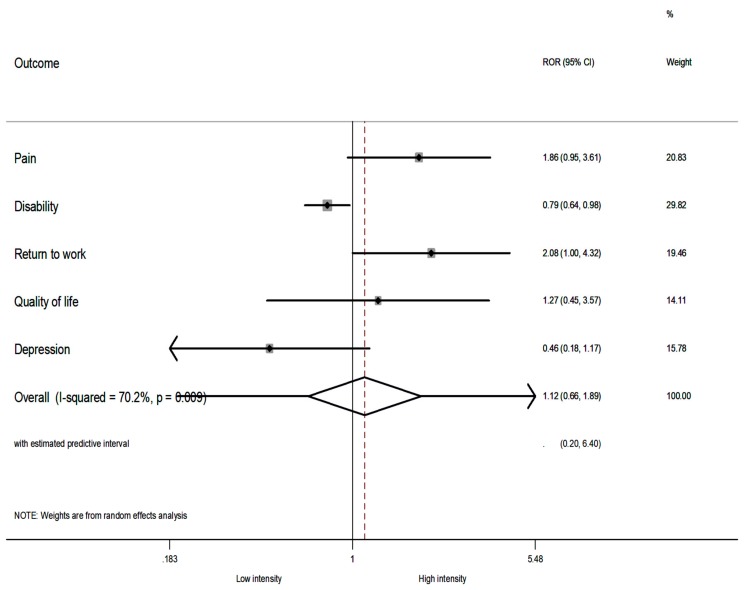
The relative odds ratios (RORs) and 95% confidence intervals (CIs) for each outcome, and the summary RORs and their 95% CIs at short term of low intensity vs. high intensity. The RORs were calculated with a random-effects model. A ROR >1 favours high intensity (i.e. >30 h per week); an ROR <1 favours low intensity (i.e. <30 h per week).

With respect to the intensity of treatment (i.e., low intensity vs. high intensity), the summary ROR was <1 only for disability, demonstrating that the IMPT was more favourable in RCTs with low intensity of treatment (i.e., RCTs with less than 30 h per week). The summary ROR was also not significant (sROR = 1.12; 95% CI 0.66–1.89; *p* = 0.672) using the random-effects model, showing that the intensity of treatment did not have an overall effect on the outcomes. Large heterogeneity was observed (I^2^ = 70%, 95% CI 21–87%) (Figure 4). Similar results were evident when the fixed-effect models were used (Figure S21, Supplementary Materials). A sensitivity analysis excluding the study of Moticone et al. (2013/2014) (References 27,28 in Supplementary Materials) did not alter the overall effects between the comparison of RORs (Figures S22–S24, Supplementary Materials).

## 4. Discussion

When evaluating the 47 RCTs of IMPT by length, contact, and intensity, we found that IMPT dosage did not have an overall influence on the reported effects in patients with non-specific CLBP. Specifically, the summary RORs were not significant when we compared the effect estimates for the investigated outcomes from the short-length and the long-length treatments and the non-daily contact with the daily contact. There was no significant influence for either intensity of the treatment. Although we found large heterogeneity between RCTs, the meta-regression analysis revealed that none of the examined factors were potential factors for heterogeneity.

Yet, per individual outcome, some evidence exhibited that IMPT of more than five weeks may be related to more “favourable” effects for pain reduction, while a long treatment with non-daily contact and low intensity may be associated with more beneficial effects for disability. These results were supported by both subgroup meta-analyses and meta-analytical comparisons of RORs between the treatment effects. This study suggests that an optimal IMPT dosage from the published work is not possible to be standardised. This finding partly agrees with the idea that the published recommended IMPT dosages are somewhat arbitrary and primarily based on clinical expertise and experience [8]. Our study, however, did not confirm an overall IMPT dose–treatment–effect association in agreement with a recently published non-inferiority RCT [9].

To our knowledge, this is the first study to examine the influence of IMPT dosage in such a systematic appraisal followed by meta-analysis and meta-epidemiological approach across the largest dataset of published RCTs, calculating the magnitude of the observed effect. Our meta-epidemiological approach with comparison of RORs is suitable for examining study design factors and characteristics on treatment effects [21,22,23]. IMPT, a complex treatment, requires a broad set-up of outcome variables. The present review had such a broad approach when evaluating IMPT dosage. Hence, we analysed dosage aspects for the individual outcomes and used a more comprehensive approach—the summary estimates of outcomes. Although IMPT dosing is rather essential in terms both of efficacy and healthcare costs [8,11], it was not systematically and thoroughly assessed in RCTs [9]. Few other studies that focused on IMPT dosage and these studies included a smaller number of RCTs or used different methodological methods and outcomes [4,10]. In a Cochrane review, Guzman et al. compared 12 IMPTs in 1964 patients with CLBP [4]. Their meta-analysis found that daily intensive IMPT (with more than 100 h per week) with respect to disability was more beneficial than monodisciplinary treatment. Evidence regarding other outcomes was either limited or ambivalent [4]. However, their review was not designed to directly study the influence of treatment dosage on outcome effects. 

Waterschoot et al. conducted a systematic review analysing the influence of IMPT dosage on disability, work, and quality of life in patients with CLBP and included 18 studies [10]. As in our study, the studies included in that review varied in terms of dosage (total duration and contact hours) and outcome effects. Their linear mixed-effect modelling showed that duration in weeks was significantly associated with the aforesaid outcomes [10]. It was somewhat surprising that we did not find an association between duration of treatment (or any other dose aspect) and effects on pain, disability, return to work, quality of life, depression, or anxiety. An explanation could be that different components such as a professional’s expertise or different types of professionals involved in an IMPT might have greater influence than the dosage variables. Waterschoot et al. also suggested that the content of such treatments is strongly related to dosage aspects; thus, the independent effect of dosage is not easily detectable [10]. A final explanation of the lack of association between and outcome effects is that there is also the possibility that the dosage does not actually influence the outcomes in this target group. The latter is supported by the subgroup analysis in Kamper et al. as they found that there was no pattern of smaller or larger effects for duration and contact with IMPT while the intensity of treatment slightly affected the treatment effects [2]. This finding is also supported by Reneman et al. who found that the dosage is not related to differences in disability [9]. 

One limitation of our study is that we did not explore the potential interaction of the role of professionals per RCT with length, contact, and intensity of treatment. Also, the individual components of IMPT may differ between RCTs without any difference in the overall dosage variables investigated here. Moreover, it is possible that some professionals might be unequally trained or have less clinical expertise for the application of the multidisciplinary treatment, and it might be hypothesised that these factors could affect the exact extent/dosage of a program. For example, well-trained professionals with extensive clinical expertise might require fewer contacts to help their patients. A related issue is the adherence to the treatment protocols within IMPT by the professionals. Many treatments require that the patients apply the achieved insights and knowledge in their home environments or jobs, and the time spent on this is usually not included in the stated doses. Furthermore, the severity of the clinical presentations of the patient groups, as well as the social context (e.g., with respect to the insurance situation) may differ substantially between RCTs; unfortunately, there are no established and standardised ways to compare chronic pain patient cohorts. It is also possible that the diversity of the professionals and the multidisciplinary nature of the treatment per se may lead to differences in treatment effects in relation to the variation of dosage [8,11]. However, there are no related data in the literature to either support or reject this hypothesis. Another limitation is that the dichotomisation between length was based on the median value of the total duration of the IMPT per week; thus, one may argue that this was somewhat subjective. However, in the literature, there is not a valid cut-off score to precisely define what is a short-length or long-length treatment, although the categorisation of contact and intensity was based on the available literature [4]. One may also argue that the overall dichotomisation of the dosage cannot accurately represent the wide spectrum of the total length in weeks, total number of hours, or the total number of contact hours per week. Indeed, our results confirm the wide variability in weeks, contact hours, and intensity as previously reported [10]. Nevertheless, the results of the meta-regression analysis using continuous variables of dosage were also not significant. In addition, large heterogeneity was found, but it cannot be explained from the dosage choices, age, gender, low risk of bias, or type of control as presented in the published RCTs. This extensive heterogeneity contributed to different results from fixed and random-effects models with respect to the comparison between length and contact of treatment, and it can be better explained by the variation in professional teams and the contents of the IMPT. Finally, we based our research on studies from a Cochrane systematic review from 2014, and, despite the additional search on PubMed, we may have missed some information.

This study does not suggest that IMPT is not beneficial for patients with CLBP. On the contrary, our results found reliable but moderate effects on pain and disability following previous evidence [1,2,3,4]. In addition, even if IMPT dosage remains controversial, the lack of association between dosing and outcome effects may mean that the rehabilitation professionals should reconsider adopting lower dosages. We also assumed that an IMPT with duration of at least five weeks with non-daily contact and low intensity would reduce pain and disability and the costs of such treatments, and increase the rehab participation of patients suffering from chronic symptomatology, thereby avoiding exhaustive and long treatments. In turn, active participation could lead to more beneficial results. Undoubtedly, CLBP is a major cause of concern globally [7,26,27] and one of the leading causes of years lived with disability [28]; therefore, optimal treatments are needed [29].

## 5. Conclusions

In this study, we showed that the IMPT dosing in general is not associated with better effects on pain, disability, work status, quality of life, depression, and anxiety in patients with non-specific CLBP. Some evidence suggests the efficacy of a long program with non-daily contact and low intensity for only pain and disability per individual outcome level, but an overall optimal dosage was not likely to be identified. This knowledge will contribute to a better evaluation from pain rehabilitation professionals to obtain insight into dosage choices that may contribute to more efficacious treatments. Further research on this topic examining also long-term outcomes is warranted. 

## Figures and Tables

**Table 1:** Characteristics of included studies.

Author, Year *	Country	Sample Size	Female %	Mean Age (or Age Range)	Treatment	Control	Definition of Chronic LBP	Total Duration (Weeks)	Contact	Contact Duration (h/Week)	RoB Assessment
Abbassi, 2012	Iran	33	88	45	IMPT	TAU	LBP >6 months	7	Non-daily contact	4	Low risk
Alaranta, 1994	Finland	293	56	41	IMPT	Physical	LBP >6 months	6	Daily contact	100	High risk
Altmaier, 1992	USA	45	73	40	IMPT	Physical	LBP >3 months	3	Daily contact	20	High risk
Basler 1997	Germany	76	76	49	IMPT	TAU	LBP >6 months	12	Non-daily contact	2.5	High risk
Bendix, 1996/1998	Denmark	106	70	40	IMPT	TAU	LBP >6 months	3	Daily contact	49	High risk
Bendix, 1995/1998	Denmark	106	75	42	IMPT	Physical	LBP >6 months	4	Daily contact	45	High risk
Bendix, 2000	Denmark	138	65	41	IMPT	Physical	LBP >6 months	4	Daily contact	45	High risk
Coole, 2013	UK	51	53	44	IMPT	Physical	LBP >3 months	16	Non-daily contact	3	High risk
Corey, 1996	Canada	138	NR	NR	IMPT	TAU	LBP >3 months	5	Daily contact	32.5	High risk
Fairbank, 2005	UK	349	51	18–55	IMPT	Surgery	LBP >1 year	3	Daily contact	75	Low risk
Harkapaa, 1989	Finland	309	37	45	IMPT	Physical	LBP for >2 years	3	Daily contact	100	High risk
Hellum, 2011	Norway	173	51	41	IMPT	Surgery	LBP >1 year	5	Non-daily contact	12	High risk
Henchoz, 2010	Switzerland	109	32	40	IMPT	Physical	LBP >3 months	3	Non-daily contact	30	High risk
Jackel, 1990	Germany	71	62	49	IMPT	WL	LBP >3 months	6	Daily contact	36	High risk
Jousset, 2004	France	84	33	40	IMPT	Physical	LBP >3 months	5	Daily contact	30	High risk
Kaapa, 2006	Finland	120	100	46	IMPT	Physical	LBP >1 year	7	Daily contact	100	Low risk
Kole-Snijders, 1999	Netherlands	148	64	40	IMPT	WL	LBP >6 months	8	Daily contact	13	High risk
Kool, 2007	Switzerland	174	21	42	IMPT	Physical	LBP >3 months	3	Non-daily contact	24	Low risk
Lambeek, 2010	Netherlands	134	42	18–65	IMPT	TAU	LBP >3 months	12	Non-daily contact	3	Low risk
Leeuw, 2008	Netherlands	85	48	45	IMPT	IMPT	LBP >3 months	16	Non-daily contact	2	Low risk
Linton, 2005	Sweden	185	83	49	IMPT	TAU	NR	6	Non-daily contact	2	High risk
Lukinmaa, 1989	Finland	158	53	44	IMPT	TAU	NR	1	Non-daily contact	2.5	High risk
Mangels, 2009	Germany	363	78	49	IMPT	Physical	ICD 10	4	Daily contact	100	Low risk
Meng, 2011	Germany	360	64	49	IMPT	TAU/IMPT	ICD 10	7	Non-daily contact	1	High risk
Mitchell, 1994	Canada	542	29	nr	IMPT	TAU	NR	8	Daily contact	35	High risk
Moix, 2003	Spain	30	53	54	IMPT	TAU	NR	11	Non-daily contact	1	High risk
Monticone, 2013	Italy	90	58	50	IMPT	TAU	LBP >3 months	5	Non-daily contact	3	Low risk
Monticone, 2014	Italy	20	NR	NR	IMPT	Physical	LBP >3 months	8	Non-daily contact	3	Low risk
Morone, 2011	Italy	73	64	60	IMPT	TAU	LBP >3 months	4	Non-daily contact	4	High risk
Morone, 2012	Italy	75	72	55	IMPT	TAU/ Physical	LBP >3 months	4	Non-daily contact	4	High risk
Nicholas, 1991	Australia	58	52	41	IMPT	Physical	LBP >6 months	5	Non-daily contact	3.5	High risk
Nicholas, 1992	Australia	20	45	44	IMPT	Physical	LBP >6 months	5	Non-daily contact	3.5	High risk
Roche, 2007/2011	France	132	35	40	IMPT	Physical	LBP >3 months	5	Daily contact	30	Low risk
Smeets, 2006/2008	Netherlands	212	42	47	IMPT	WL	LBP >3 months	10	Non-daily contact	7.1	Low risk
Skouen, 2002	Norway	195	44	43	IMPT	TAU/IMPT	NR	4	Daily contact	30	High risk
Schweikert, 2006	Germany	409	17	47	IMPT	Physical	LBP >6 months	3	Daily contact	17.5	High risk
Strand, 2001	Norway	117	61	43	IMPT	TAU	ICPC diagnosis	5	Daily contact	30	High risk
Streibelt, 2009	Germany	222	17	46	IMPT	Physical	NR	3	Non-daily contact	20	High risk
Tavafian, 2008	Iran	102	100	43	IMPT	TAU	LBP >3 months	1	Non-daily contact	5	High risk
Tavafian, 2011	Iran	197	22	45	IMPT	TAU	LBP >3 months	1	Non-daily contact	10	Low risk
Tavafian, 2014	Iran	178	75	44	IMPT	TAU	LBP >3 months	1	Daily contact	5	High risk
Tavafian 2017	Iran	146	78	46	IMPT	TAU	LBP >3 months	1	Daily contact	5	Low risk
Tavafian, 2017	Iran	165	79	45	IMPT	TAU	LBP >3 months	1	Daily contact	5	Low risk
Turner, 1990	USA	96	49	44	IMPT	Physical/ WL	LBP >6 months	8	Non-daily contact	2	High risk
Van den Hout, 2003	Netherlands	84	34	40	IMPT	IMPT	LBP >6 months	8	Non-daily contact	20	High risk
Vollenbroek-Hutten, 2004	Netherlands	163	NR	39	IMPT	TAU	LBP >6 months	7	Non-daily contact	9	Low risk
Von Korff, 2005	USA	240	63	50	IMPT	TAU	score >7/23 on RMDQ	1	Non-daily contact	3	High risk

* See references of included randomised controlled trials (RCTs) 1–47 in Supplementary Materials; ICD 10—International Statistical Classification of Diseases and Related Health Problems, Tenth Revision, ICPC—International Classification of Primary Care, LBP—lower back pain, IMPT—interdisciplinary multimodal pain therapy, NR—not reported, RMDQ—Roland Morris Disability Questionnaire, RoB—risk of bias, TAU—treatment as usual, UK—United Kingdom, USA—United States of America, WL—waiting list.

**Table 2:** Characteristics and subgroup meta-analysis for dose of IMPT by length, contact, and intensity of the six investigated outcomes.

Short-Term Outcomes	No. of RCTs	Average Total Duration (Median Weeks, IQR)	Average h/Week (Median, IQR)	Level of Daily/Non-Daily Contact (*n*)	Level of Active (i.e., Physical)/Non-Active Control (i.e., WL/TAU) (*n*)	Level of Low Risk/High Risk of Bias (*n*)	Overall ES (95% CI) Random-Effects Model	*p*-Value	I^2^ (%; 95% CI) *p*-Value
**Outcome 1: Pain**									
Length									
Short length (<5 weeks)	12	3 (1–4)	7.5 (4–22)	8/4	6/6	3/9	SMD, −0.33 (−0.55 to −0.11)	0.003	81 (67–88) 0.000
Long length (≥5 weeks)	16	8 (5.5–10)	3.5 (2.7–10)	4/12	8/8	8/8	SMD, −0.45 (−0.73 to −0.17)	0.001	79 (64–85) 0.000
Contact									
Non-daily contact	16	7.5 (4.5–10)	3.3 (2.7–4)	0/16	7/9	6/10	SMD, −0.50 (−0.79 to −0.20)	0.001	80 (67–86) 0.000
Daily contact	12	3 (2–5.5)	22 (11.5–68)	12/0	7/5	5/7	SMD, -0.29 (−0.49 to −0.09)	0.005	78 (59–86) 0.000
Intensity									
<30 h per week	23	5 (3–8)	4 (3–7.1)	7/16	10/13	8/15	SMD, −0.42 (−0.62 to −0.20)	<0.000	81 (71–86) 0.000
>30 h per week	5	5 (4–6)	100 (36–100)	5/0	4/1	3/2	SMD, −0.32 (−0.63 to −0.05)	0.022	79 (31–89) 0.001
**Outcome 2: Disability**									
Length									
Short length (<5 weeks)	10	3 (1–4)	7.5 (4–30)	5/5	5/5	2/8	SMD, −0.27 (−0.48 to −0.07)	0.007	75 (47–85) 0.000
Long length (≥5 weeks)	17	7 (6–10)	3.5 (3–9)	4/13	9/8	9/8	SMD, −0.51 (−0.78 to −0.24)	<0.000	82 (73–88) 0.000
Contact									
Non-daily contact	18	7 (4–10)	3.5 (3–4)	0/18	8/10	7/11	SMD, −0.58 (−0.86 to −0.31)	<0.000	81 (70–87) 0.000
Daily contact	9	4 (1–3)	36 (18–100)	9/0	6/3	4/5	SMD, −0.16 (−0.33–0.01)	0.055	67 (16–82) 0.002
Intensity									
<30 h per week	20	6 (4–9)	3.8 (3–6)	3/17	8/12	8/12	SMD, −0.49 (−0.74 to −0.24)	<0.000	85 (78–89) 0.000
>30 h per week	7	5 (3–6)	100 (30–100)	6/1	6/1	3/4	SMD, −0.26 (−0.43 to −0.09)	0.003	54 (0–78) 0.043
**Outcome 3: Return to work**									
Length									
Short length (<5 weeks)	3	3 (1–4)	24 (3–30)	2/1	1/2	1/2	OR, 1.46 (0.82–2.62)	0.199	42 (0–83) 0.177
Long length (≥5 weeks)	2	5 (5–5)	30 (30–30)	2/0	2/0	1/1	OR, 1.10 (0.55–2.20)	0.786	0 (NA) ^#^ 0.938
Contact									
Non-daily contact	1	1 (1–1)	3 (3–3)	0/1	0/1	0/1	OR, 0.91 (0.31–2.68)	0.864	NA
Daily contact	4	4.5 (3.5–5)	30 (27–30)	4/0	1/3	2/2	OR, 1.46 (0.96–2.21)	0.075	12 (0–72) 0.332
Intensity									
<30 h per week	2	2 (1–3)	13.5 (3–24)	1/1	1/1	1/1	OR, 1.63 (0.65–4.09)	0.297	56 (NA) ^#^ 0.133
>30 h per week	3	5 (4–5)	30 (30–30)	3/0	2/1	1/2	OR, 1.12 (0.69–1.82)	0.645	0 (0–73) 0.994
**Outcome 4: Quality of life**									
Length									
Short length (<5 weeks)	8	1.5 (1–3.5)	7.5 (4–13.7)	5/3	3/5	2/6	SMD, 0.49 (0.14–0.84)	0.006	83 (65–90) 0.000
Long length (≥5 weeks)	1	10 (10–10)	7.1 (7.1–7.1)	0/1	1/0	1/0	SMD, 0.14 (−0.24–0.52)	0.470	NA
Contact									
Non-daily contact	4	4 (1–10)	4 (3–7.1)	0/4	2/2	2/2	SMD, 0.53 (0.09–0.98)	0.019	64 (0–86) 0.038
Daily contact	5	1.5 (1–3)	10 (5–18)	5/0	2/3	1/4	SMD, 0.38 (−0.06–0.81)	0.089	88 (70–93) 0.000
Intensity									
<30 h per week	8	2 (1–4))	6 (4–10)	4/4	3/5	2/6	SMD, 0.54 (0.25–0.83)	<0.000	75 (38–86) 0.000
>30 h per week	1	2 (2–2)	100 (100–100)	1/0	1/0	1/0	SMD, −0.38 (−0.74 to −0.02)	0.041 *	NA
**Outcome 5: Depression**									
Length									
Short length (<5 weeks)	2	3.5 (3–4)	58.8 (17.5–100)	2/0	2/0	1/1	SMD, 0.08 (−0.22–0.39)	0.584	71 (NA)^#^ 0.063
Long length (≥5 weeks)	8	7.5 (5.5–9)	5.3 (2.8–21.5)	2/6	5/3	3/5	SMD, −0.09 (−0.29–0.11)	0.358	20 (0–64) 0.273
Contact									
Non-daily contact	6	8 (5–10)	3.5 (2–7.1)	0/6	4/2	2/4	SMD, 0.01 (−0.21–0.22)	0.959	0 (0–61) 0.562
Daily contact	4	5 (3.5–6.5)	68 (26.8–100)	4/0	3/1	2/2	SMD, −0.07 (−0.35–0.22)	0.653	72 (0–88) 0.013
Intensity									
<30 h per week	7	8 (5–10)	7.1 (2–17.5)	2/5	4/3	2/5	SMD, 0.12 (−0.03–0.27)	0.119	1 (0–59) 0.414
>30 h per week	3	5 (4–7)	100 (3.5–100)	2/1	3/0	2/1	SMD, −0.18 (−0.46–0.10)	0.202	46 (0–84) 0.155
**Outcome 6: Anxiety**									
Length									
Short length (<5 weeks)	1	3 (3–3)	17.5 (17.5–17.5)	1/0	1/0	0/1	SMD, 0.08 (−0.13–0.29)	0.455	NA
Long length (≥5 weeks)	1	5 (5–5)	3.5 (3.5–3.5)	0/1	1/0	0/1	SMD, −0.58 (−1.48–0.32)	0.209	NA
Contact									
Non-daily contact	1	5 (5–5)	3.5 (3.5–3.5)	0/1	1/0	0/1	SMD, −0.58 (−1.48–0.32)	0.209	NA
Daily contact	1	3 (3–3)	17.5 (17.5–17.5)	1/0	1/0	0/1	SMD, 0.08 (−0.13–0.29)	0.455	NA
Intensity									
<30 h per week	2	4 (3–5)	10.5 (3.5–17.5)	1/1	2/0	0/2	SMD, −0.10 (−0.67–0.48)	0.740	48 (NA) ^#^ 0.164
>30 h per week	0	0 (0–0)	0 (0–0)	0/0	0/0	0/0	NA	NA	NA

Notes: * favours control, ^#^ degrees of freedom (df *n* − 1) must be at least 2, CI—confidence interval, ES—effect size, OR—odds ratio, I^2^—I square metric of heterogeneity, IQR—interquartile range, TAU—treatment as usual, SMD—standardised mean difference, NA—not applicable, WL—waiting list.

## Supplementary Materials

The following are available online at https://www.mdpi.com/2077-0383/8/6/871/s1. Figure S1: Study flow diagram, Figure S2: Funnel plot for the pain outcome. Egger’s test for publication bias was not significant (*p* = 0.141), Figure S3: Funnel plot for the disability outcome. Egger’s test for publication bias was significant (*p* = 0.018), Figure S4: Funnel plot for the return to work outcome. Egger’s test for publication bias was not significant (*p* = 0.141), Figure S5: Funnel plot for the quality of life outcome. Egger’s test for publication bias was significant (*p* = 0.060), Figure S6: Funnel plot for the depression outcome. Egger’s test for publication bias was significant (*p* = 0.081), Figure S7: Sensitivity analysis by length for the pain outcome, after excluding the study of Moticone et al. (2013/2014), Figure S8: Sensitivity analysis by contact for the pain outcome, after excluding the study of Moticone et al. (2013/2014), Figure S9: Sensitivity analysis by intensity for the pain outcome, after excluding the study of Moticone et al. (2013/2014), Figure S10: Sensitivity analysis by length for the disability outcome, after excluding the study of Moticone et al. (2013/2014), Figure S11: Sensitivity analysis by contact for the disability outcome, after excluding the study of Moticone et al. (2013/2014), Figure S12: Sensitivity analysis by intensity for the disability outcome, after excluding the study of Moticone et al. (2013/2014), Figure S13: Forest plot for the pain outcome all studies included, Figure S14: Forest plot for the disability outcome all studies included, Figure S15: Forest plot for the return to work outcome all studies included, Figure S16: Forest plot for the quality of life outcome all studies included, Figure S17: Forest plot for the depression outcome all studies included, Figure S18: Forest plot for the anxiety outcome all studies included, Figure S19: The relative odds ratios (RORs) and 95% confidence intervals (CIs) for each outcome at short term of a short-length treatment vs. long-length treatment. The RORs were calculated with a fixed-effect model. A ROR >1 favours long length; a ROR <1 favours short length, Figure S20: The relative odds ratios (RORs) and 95% confidence intervals (CIs) for each outcome at short term of a non-daily contact vs. daily contact. The RORs were calculated with a fixed-effect model. A ROR >1 favours daily contact; a ROR <1 favours non-daily contact, Figure S21: The relative odds ratios (RORs) and 95% confidence intervals (CIs) for each outcome at short term of a low intensity vs. high intensity. The RORs were calculated with a fixed-effect model. A ROR >1 favours high intensity (i.e., >30 h per week); a ROR <1 favours low intensity (i.e., <30 h per week), Figure S22: The relative odds ratios (RORs) and 95% confidence intervals (CIs) for each outcome at short term of a short-length treatment vs. long-length treatment, after excluding the study of Moticone et al. (2013/2014). The RORs were calculated with a random-effects model. A ROR >1 favours long length; a ROR <1 favours short length, Figure S23: The relative odds ratios (RORs) and 95% confidence intervals (CIs) for each outcome at short term of a non-daily contact vs. daily contact, after excluding the study of Moticone et al. (2013/2014). The RORs were calculated with a random-effects model. A ROR >1 favours daily contact; a ROR <1 favours non-daily contact, Figure S24: The relative odds ratios (RORs) and 95% confidence intervals (CIs) for each outcome at short term of a low intensity vs. high intensity, after excluding the study of Moticone et al. (2013/2014). The RORs were calculated with a random-effects model. A ROR >1 favours high intensity (i.e., >30 h per week); a ROR <1 favours low intensity (i.e., <30 h per week), Table S1: Risk of bias (RoB) according to the Cochrane Back Review Group criteria, Table S2: Results of the meta-regression analyses of potential moderators of the six examined outcomes, Table S3: Checklist summarizing compliance with PRISMA guidelines, Box S1: Search details in PubMed, Box S2: References of included RCTs.
