# Supplementary material for: A Meta-Epidemiological Appraisal of the Effects of Interdisciplinary Multimodal Pain Therapy Dosing for Chronic Low Back Pain
Source: J Clin Med. 2019 Jun 18;8(6):871. doi: 10.3390/jcm8060871 (PMC6616996; doi:10.3390/jcm8060871)
Supplement: Supplementary file 1 [file jcm-08-00871-s001.pdf]

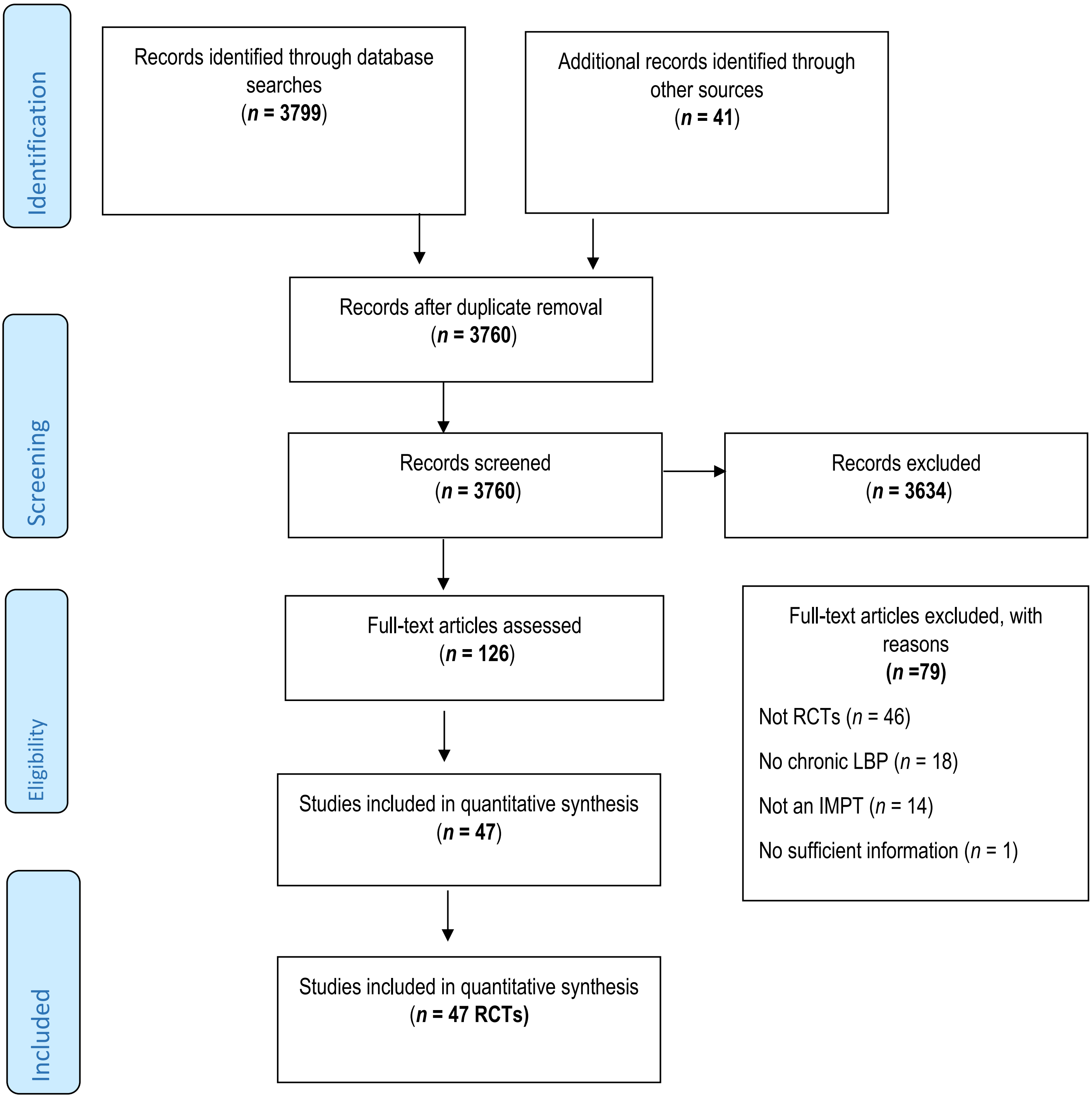

Figure S1. Study flow diagram.

Notes: RCTs = randomized controlled trials, LBP = low back pain, IMPT = interdisciplinary multimodal pain therapy.

Box S1. Search details in PubMed.

(("low back pain"[MeSH Terms] OR ("low"[All Fields] AND "back"[All Fields] AND "pain"[All Fields]) OR "low back pain"[All Fields]) OR (chronic[All Fields] AND ("low back pain"[MeSH Terms] OR ("low"[All Fields] AND "back"[All Fields] AND "pain"[All Fields]) OR "low back pain"[All Fields])) OR LBP[All Fields] OR CLBP[All Fields] OR ("sciatica"[MeSH Terms] OR "sciatica"[All Fields]) OR ("low back pain"[MeSH Terms] OR ("low"[All Fields] AND "back"[All Fields] AND "pain"[All Fields]) OR "low back pain"[All Fields] OR "lumbago"[All Fields]) OR ("spondylosis"[MeSH Terms] OR "spondylosis"[All Fields])) AND ((("pain"[MeSH Terms] OR "pain"[All Fields]) AND ("rehabilitation"[Subheading] OR "rehabilitation"[All Fields] OR "rehabilitation"[MeSH Terms])) OR ("pain management"[MeSH Terms] OR ("pain"[All Fields] AND "management"[All Fields]) OR "pain management"[All Fields] OR ("pain"[All Fields] AND "therapy"[All Fields]) OR "pain therapy"[All Fields]) OR ("combined modality therapy"[MeSH Terms] OR ("combined"[All Fields] AND "modality"[All Fields] AND "therapy"[All Fields]) OR "combined modality therapy"[All Fields]) OR ("interdisciplinary studies"[MeSH Terms] OR ("interdisciplinary"[All Fields] AND "studies"[All Fields]) OR "interdisciplinary studies"[All Fields] OR "multidisciplinary"[All Fields]) OR ("interdisciplinary studies"[MeSH Terms] OR ("interdisciplinary"[All Fields] AND "studies"[All Fields]) OR "interdisciplinary studies"[All Fields] OR "interdisciplinary"[All Fields]) OR multimodal[All Fields] OR (("pain"[MeSH Terms] OR "pain"[All Fields]) AND program[All Fields] AND team[All Fields] AND based[All Fields] AND ("rehabilitation"[Subheading] OR "rehabilitation"[All Fields] OR "rehabilitation"[MeSH Terms])) OR vocational[All Fields]) AND (("randomized controlled trial"[Publication Type] OR "randomized controlled trials as topic"[MeSH Terms] OR "randomized controlled trials"[All Fields] OR "randomised controlled trials"[All Fields]) OR (random[All Fields] OR random'[All Fields] OR random1[All Fields] OR random1y[All Fields] OR randomaized[All Fields] OR randomally[All Fields] OR randomaly[All Fields] OR randomamplified[All Fields] OR randoman[All Fields] OR randomand[All Fields] OR randomate[All Fields] OR randombalance[All Fields] OR randombased[All Fields] OR randomboost[All Fields] OR randombred[All Fields] OR randombreds[All Fields] OR randomc[All Fields] OR randomcoil[All Fields] OR randomcommittee[All Fields] OR randomcontrol[All Fields] OR randomdata[All Fields] OR randomdigit[All Fields] OR randomdock[All Fields] OR randomdot[All Fields] OR randomdouble[All Fields] OR randome[All Fields] OR randomed[All Fields] OR randomeffect[All Fields] OR randomeffects[All Fields] OR randomely[All Fields] OR randomer[All Fields] OR randomesque[All Fields] OR randomezed[All Fields] OR randomferns[All Fields] OR randomforest[All Fields] OR randomforest'[All Fields] OR randomforest4life[All Fields] OR randomforests[All Fields] OR randomforestsrc[All Fields] OR randomforrest[All Fields] OR randomfrog[All Fields] OR randomglm[All Fields] OR randomi[All Fields] OR randomiazed[All Fields] OR randomic[All Fields] OR randomically[All Fields] OR randomicaly[All Fields] OR randomiced[All Fields] OR randomicity[All Fields] OR randomico[All Fields] OR randomided[All Fields] OR randomied[All Fields] OR randomifzed[All Fields] OR randomil[All Fields] OR randomily[All Fields] OR randomin[All Fields] OR randomined[All Fields] OR randomingly[All Fields] OR randominization[All Fields] OR randominzed[All Fields] OR randomirrespective[All Fields] OR randomis[All Fields] OR randomisable[All Fields] OR randomisaion[All Fields] OR randomisatie[All Fields] OR randomisation[All Fields] OR randomisation'[All Fields] OR randomisations[All Fields] OR randomisationsecondary[All Fields] OR

randomisationsentscheidung[All Fields] OR randomisaton[All Fields] OR randomisd[All Fields] OR randomise[All Fields] OR randomised[All Fields] OR randomised'[All Fields] OR randomisedbhc[All Fields] OR randomisedclinical[All Fields] OR randomisedcontrolled[All Fields] OR randomisedrandomised[All Fields] OR randomisee[All Fields] OR randomisees[All Fields] OR randomisely[All Fields] OR randomiser[All Fields] OR randomisera[All Fields] OR randomiserad[All Fields] OR randomiserade[All Fields] OR randomiseras[All Fields] OR randomiserede[All Fields] OR randomiseren[All Fields] OR randomiseret[All Fields] OR randomisering[All Fields] OR randomiseringen[All Fields] OR randomiseringens[All Fields] OR randomiseringsproblemet[All Fields] OR randomisert[All Fields] OR randomiserte[All Fields] OR randomiserten[All Fields] OR randomises[All Fields] OR randomisieren[All Fields] OR randomisierlen[All Fields] OR randomisiert[All Fields] OR randomisierte[All Fields] OR randomisiertekontrollierte[All Fields] OR randomisierten[All Fields] OR randomisierter[All Fields] OR randomisierung[All Fields] OR randomisierungs[All Fields] OR randomisierungsbasis[All Fields] OR randomisierungslisten[All Fields] OR randomisierungsstudie[All Fields] OR randomisierungsverfahren[All Fields] OR randomising[All Fields] OR randomisized[All Fields] OR randomisly[All Fields] OR randomisons[All Fields] OR randomiz[All Fields] OR randomizability[All Fields] OR randomizable[All Fields] OR randomizacao[All Fields] OR randomizace[All Fields] OR randomizaci[All Fields] OR randomizacja[All Fields] OR randomizad[All Fields] OR randomizada[All Fields] OR randomizadas[All Fields] OR randomizadely[All Fields] OR randomizado[All Fields] OR randomizados[All Fields] OR randomizaion[All Fields] OR randomizalt[All Fields] OR randomizar[All Fields] OR randomizat[All Fields] OR randomization[All Fields] OR randomization'[All Fields] OR randomization's[All Fields] OR randomizationin[All Fields] OR randomizations[All Fields] OR randomizations'[All Fields] OR randomizationstudies[All Fields] OR randomizato[All Fields] OR randomizatsii[All Fields] OR randomizd[All Fields] OR randomize[All Fields] OR randomize'[All Fields] OR randomized[All Fields] OR randomized'[All Fields] OR randomized150[All Fields] OR randomizedcontrolled[All Fields] OR randomizedcontrolledclinicaltrialstif20[All Fields] OR randomizedcontrolledtrial[All Fields] OR randomizedcontrolledtrials[All Fields] OR randomizedcontroltrials[All Fields] OR randomizedcrossover[All Fields] OR randomizeddouble[All Fields] OR randomizedduring[All Fields] OR randomizedinto[All Fields] OR randomizedly[All Fields] OR randomizedphase[All Fields] OR randomizedsequence[All Fields] OR randomizedto[All Fields] OR randomizedtrial[All Fields] OR randomizely[All Fields] OR randomizer[All Fields] OR randomizer'[All Fields] OR randomizers[All Fields] OR randomizes[All Fields] OR randomizied[All Fields] OR randomizing[All Fields] OR randomizing'[All Fields] OR randomizingly[All Fields] OR randomiziranih[All Fields] OR randomiziranim[All Fields] OR randomizirano[All Fields] OR randomiziranoj[All Fields] OR randomiziranom[All Fields] OR randomizirovannoe[All Fields] OR randomizirovannogo[All Fields] OR randomizirovannye[All Fields] OR randomizirovannykh[All Fields] OR randomizirovannym[All Fields] OR randomizovana[All Fields] OR randomizovane[All Fields] OR randomizovanej[All Fields] OR randomizovany[All Fields] OR randomizovanych[All Fields] OR randomizowana[All Fields] OR randomizowane[All Fields] OR randomizowanegobadania[All Fields] OR randomizowanych[All Fields] OR randomizzata[All Fields] OR randomizzate[All Fields] OR randomizzati[All Fields] OR randomizzato[All Fields] OR randomizzazione[All Fields] OR randomizziamo[All Fields] OR randomjungle[All Fields] OR randoml[All Fields] OR randomlike[All Fields] OR randomline[All Fields] OR randomlly[All Fields] OR randomly[All Fields] OR randomly'[All Fields] OR randomly'assign[All Fields] OR randomlyallocated[All Fields] OR randomlyand[All Fields] OR randomlyassigned[All Fields] OR randomlychosen[All Fields] OR randomlydivided[All Fields] OR randomlyequally[All Fields] OR randomlyinserted[All Fields] OR randomlyn[All Fields] OR randomlyselected[All Fields] OR randomlysplit[All Fields] OR randomlyuniformly[All Fields] OR randommess[All Fields] OR randommethacrylic[All Fields]

OR randommized[All Fields] OR randommobility[All Fields] OR randomnes[All Fields] OR randomness[All Fields] OR randomness'[All Fields] OR randomnesses[All Fields] OR randomnicity[All Fields] OR randomniized[All Fields] OR randomnized[All Fields] OR randomnly[All Fields] OR randomomized[All Fields] OR randomosed[All Fields] OR randomoutcross[All Fields] OR randompairs[All Fields] OR randompattern[All Fields] OR randompf[All Fields] OR randomphase[All Fields] OR randompod[All Fields] OR randompolypeptides[All Fields] OR randompower[All Fields] OR randoms[All Fields] OR randoms'[All Fields] OR randomsample[All Fields] OR randomsamples[All Fields] OR randomsampling[All Fields] OR randomsed[All Fields] OR randomselection[All Fields] OR randomsequences[All Fields] OR randomsied[All Fields] OR randomsource[All Fields] OR randomspot[All Fields] OR randomstudie[All Fields] OR randomsurvivalforest[All Fields] OR randomtree[All Fields] OR randomvariables[All Fields] OR randomwalk[All Fields] OR randomwalkrestartmh[All Fields] OR randomwalksat[All Fields] OR randomy[All Fields] OR randomyl[All Fields] OR randomyly[All Fields] OR randomzed[All Fields] OR randomzied[All Fields] OR randomzing[All Fields] OR randomzorgtoewijzing[All Fields]) AND (controlled[All Fields] AND ("Trials"[Journal] OR "trials"[All Fields])) OR ("randomized controlled trial"[Publication Type] OR "randomized controlled trials as topic"[MeSH Terms] OR "randomised controlled trials"[All Fields] OR "randomized controlled trials"[All Fields]))

Table S1. Risk of bias (RoB) according to the Cochrane Back Review Group criteria.

Author, year	Random sequence generation	Allocation concealment	Blinding of participants	Blinding of clinicians	Blinding of outcome assessment	Incomplete outcome data	Intention to treat analysis	Selective reporting	Comparability of groups at baseline	Compliance	Cointerventions	Timing of assessment	RoB Assessment
**Abbassi, 2012**	1	2	3	3	3	1	1	2	1	1	2	1	Low risk
**Alaranta, 1994**	2	2	3	3	3	1	2	2	1	2	2	1	High risk
**Basler, 1997**	1	2	3	3	3	3	2	2	1	2	2	1	High risk
**Bendix, 1996/1998**	1	2	3	3	3	1	2	2	1	2	2	1	High risk
**Bendix, 1995/1998**	1	2	3	3	3	1	2	2	1	1	2	2	High risk
**Bendix, 2000**	1	2	3	3	3	1	1	2	1	2	2	1	High risk
**Coole, 2013**	2	2	3	3	3	3	2	2	1	3	2	1	High risk
**Fairbank, 2005**	1	2	3	3	3	1	1	2	1	2	1	1	Low risk
**Harkapaa, 1989**	2	2	3	3	3	1	2	2	1	3	2	1	High risk
**Hellum, 2011**	1	1	3	3	3	1	1	2	3	2	2	1	High risk
**Henchoz, 2010**	1	1	3	3	3	3	1	2	1	2	2	1	High risk
**Jackel, 1990**	2	2	3	3	3	2	2	2	1	2	2	1	High risk
**Jousset, 2004**	1	2	3	3	3	1	2	2	1	2	2	1	High risk
**Kaapa, 2006**	1	1	3	3	3	1	1	2	1	2	2	1	Low risk
**Kole-Snijders, 1999**	1	1	3	3	3	3	1	2	3	1	2	1	High risk
**Kool, 2007**	1	2	3	3	3	1	1	2	1	1	1	1	Low risk
**Lambeek, 2010**	1	1	3	3	3	1	1	3	1	3	2	1	Low risk
**Leeuw, 2008**	1	1	3	3	3	1	1	2	1	3	1	1	Low risk
**Linton, 2005**	1	2	3	3	3	3	1	2	1	1	3	1	High risk
**Lukinmaa, 1989**	2	2	3	3	3	1	2	2	1	2	2	1	High risk
**Mangels, 2009**	1	1	3	3	3	1	1	2	1	2	2	1	Low risk
**Meng, 2011**	1	1	3	3	3	1	2	2	1	2	2	1	High risk
**Mitchell, 1994**	2	2	3	3	3	2	2	2	2	2	2	1	High risk

**Moix, 2003**	1	2	3	3	3	1	2	2	1	2	2	1	High risk
**Monticone, 2013**	1	1	3	3	3	1	1	2	1	2	2	1	Low risk
**Morone, 2011**	2	1	3	3	3	1	3	2	1	2	2	1	High risk
**Morone, 2012**	1	1	3	3	3	1	1	2	2	2	2	1	High risk
**Nicholas, 1991**	2	1	3	3	3	3	2	2	2	2	2	1	High risk
**Nicholas, 1992**	2	1	3	3	3	1	2	2	2	2	2	1	High risk
**Roche, 2007/2011**	1	1	3	3	3	1	2	2	1	1	2	1	Low risk
**Schweikert, 2006**	1	1	3	3	3	3	2	2	2	2	2	1	High risk
**Skouen, 2002**	1	1	3	3	3	1	2	2	2	2	1	1	High risk
**Smeets, 2006/2008**	1	1	3	3	3	1	1	2	1	1	1	1	Low risk
**Strand, 2001**	1	1	3	3	3	3	2	2	1	2	2	1	High risk
**Streibelt, 2009**	1	1	3	3	3	3	1	2	3	2	2	1	High risk
**Tavafian 2008**	2	3	3	3	3	3	2	2	1	2	1	1	High risk
**Tavafian 2011**	1	1	3	3	3	1	3	1	1	2	2	1	Low risk
**Turner, 1990**	2	2	3	3	3	3	3	2	1	2	2	1	High risk
**Van den Hout, 2003**	1	1	3	3	3	3	2	2	3	2	2	1	High risk
**Vollenbroek-Hutten, 2004**	1	1	3	3	3	1	2	2	1	2	1	1	Low risk
**Von Korff, 2005**	2	2	3	3	3	1	2	2	1	2	2	1	High risk
**Tavafian 2017**	1	1	3	3	3	1	3	1	1	2	2	1	Low risk
**Tavafian 2017**	1	1	3	3	3	1	3	1	1	2	2	1	Low risk
**Tavafian 2014**	2	3	3	3	3	3	2	2	1	2	1	1	High risk
**Monticone, 2014**	1	1	3	3	3	1	1	2	1	2	2	1	Low risk
**Altmaier, 1992**	3	3	3	3	3	1	3	1	1	3	1	1	High risk
**Corey, 1996**	2	1	1	3	3	3	3	1	1	2	2	1	High risk

Ratings of "1" indicate the study has a low RoB on that criteria; ratings of "2" indicate uncertain RoB on that criteria; ratings of "3" indicate high RoB on that criteria; RoB = risk of bias. Studies satisfying at least six of the 12 criteria and having no having no serious flaws (e.g., 80% drop-out rate in one group) were considered as "low" risk of bias. Studies with serious flaws, or those in which fewer than six of the criteria are met were considered as having a "high" risk of bias.

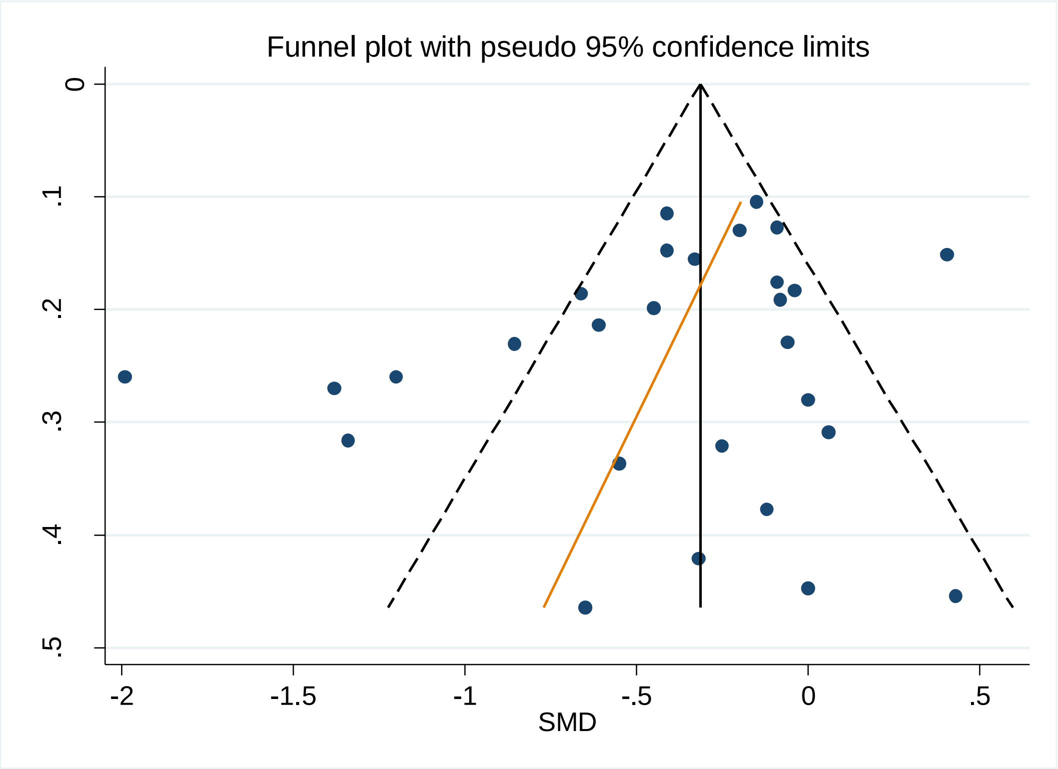

Figure S2. Funnel plot for the pain outcome. Egger's test for publication bias was not significant (p = 0.141).

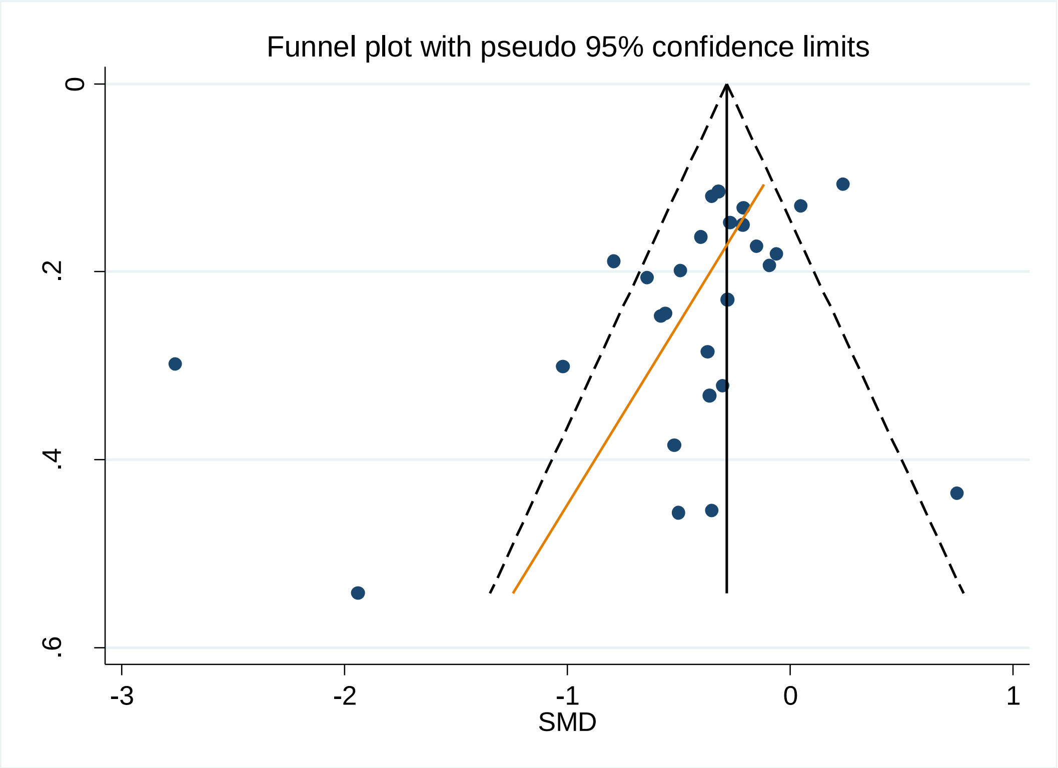

Figure S3. Funnel plot for the disability outcome. Egger's test for publication bias was significant (p = 0.018).

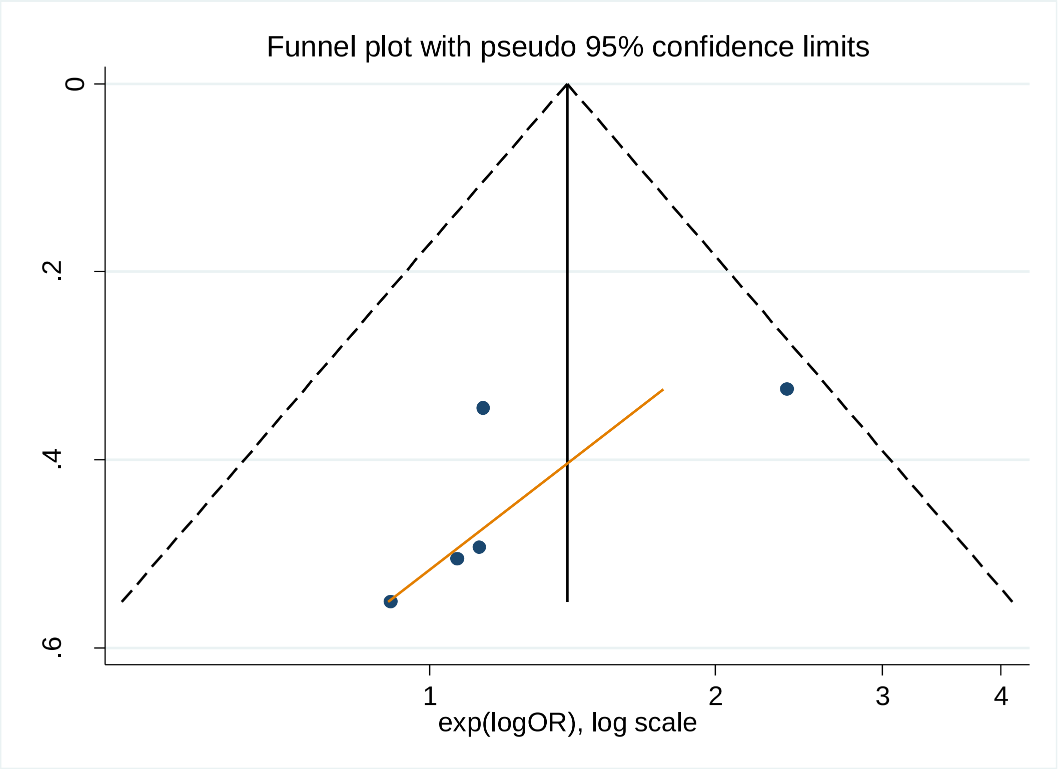

Figure S4. Funnel plot for the return to work outcome. Egger's test for publication bias was not significant (p = 0.141).

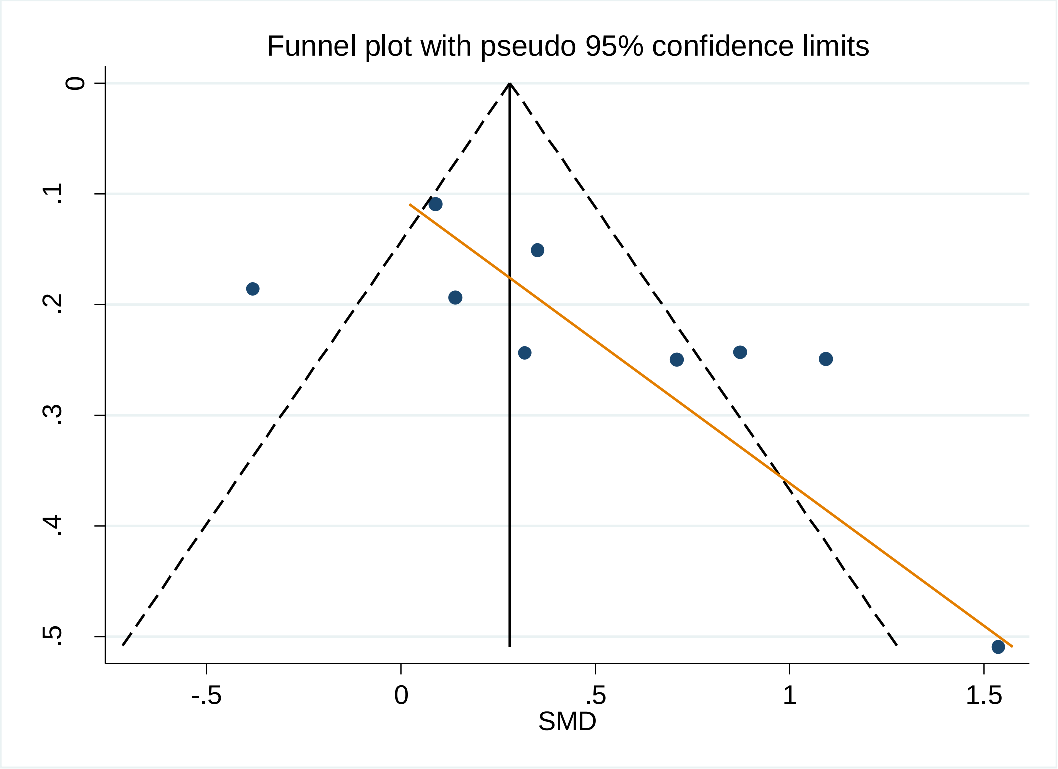

Figure S5. Funnel plot for the quality of life outcome. Egger's test for publication bias was significant (p = 0.060).

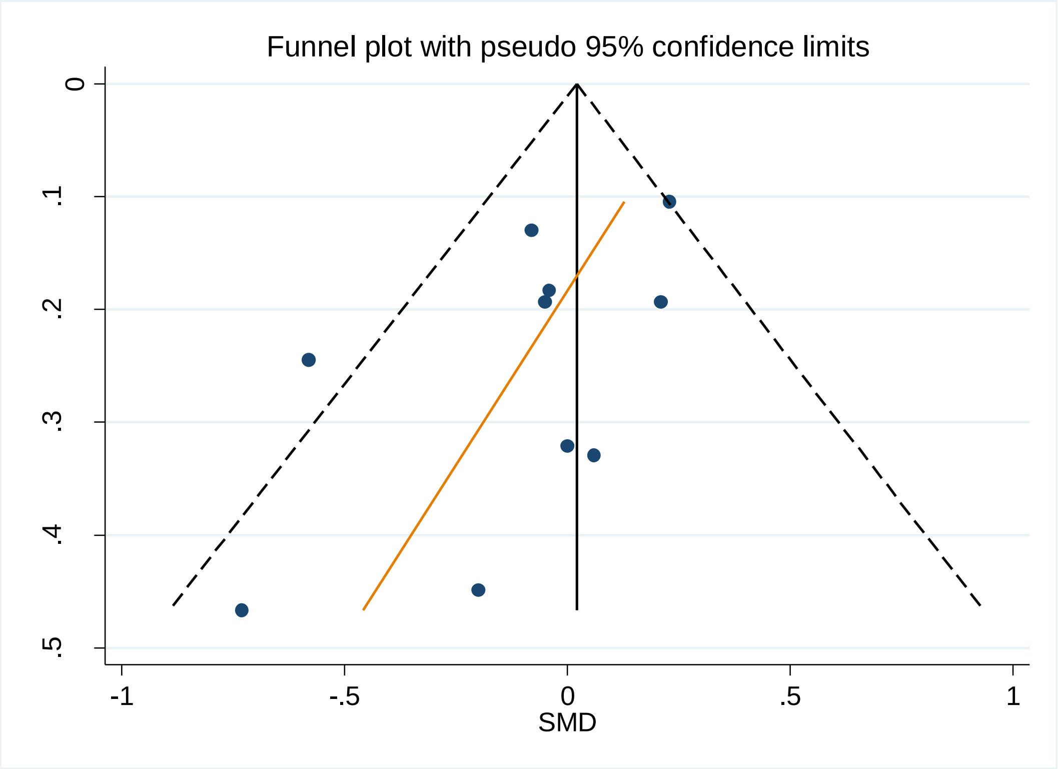

Figure S6. Funnel plot for the depression outcome. Egger's test for publication bias was significant (p = 0.081).

Table S2. Results of the meta-regression analyses of potential moderators of the six examined outcomes.

	Estimate	SE	t	p-value
**Outcome 1: Pain**				
Length of treatment (total duration in weeks)	−0.27	0.33	−0.81	0.430
Contact (daily contact)	−0.02	0.41	−0.05	0.963
Intensity (total hours per week)	−0.20	0.44	−0.45	0.655
Age	−0.04	0.03	−1.31	0.207
Gender (female)	0.33	0.29	1.13	0.273
Type of Control (active)	0.25	0.26	0.26	0.336
RoB assessment (low risk of bias)	−0.00	0.20	−0.01	0.996
**Outcome 2: Disability**				
Length of treatment (total duration in weeks)	−0.02	0.04	−0.37	0.719
Contact (daily contact)	−0.01	0.01	−1.28	0.218
Intensity (total hours per week)	0.01	0.01	1.44	0.168
Age	−0.02	0.03	−0.73	0.475
Gender (female)	0.92	0.55	1.65	0.118
Type of Control (active)	−0.13	0.31	−0.42	0.679
RoB assessment (low risk of bias)	−0.12	0.24	−0.51	0.613
**Outcome 3: Return to work**				
Length of treatment (total duration in weeks)	−0.04	0.17	−0.23	0.830
Contact (daily contact)	−0.01	0.01	−0.41	0.709
Intensity (total hours per week)	0.00	0.02	0.23	0.832
Age	−0.03	0.07	−0.50	0.653
Gender (female)	0.30	0.21	1.47	0.216
Type of Control (active)	0.09	0.46	0.20	0.862
RoB assessment (low risk of bias)	0.55	0.37	1.48	0.235
**Outcome 4: Quality of life**				
Length of treatment (total duration in weeks)	−0.06	0.06	−0.97	0.363
Contact (daily contact)	−0.01	0.00	−2.31	0.054
Intensity (total hours per week)	−0.01	0.00	−2.19	0.065
Age	0.01	0.03	0.25	0.816
Gender (female)	0.24	0.46	0.53	0.617
Type of Control (active)	−0.47	0.47	−1.00	0.356
RoB assessment (low risk of bias)	−0.33	0.40	−0.82	0.437
**Outcome 5: Depression**				

Length of treatment (total duration in weeks)	−0.10	.06	−1.61	0.249
Contact (daily contact)	0.00	0.00	2.25	0.154
Intensity (total hours per week)	−0.09	0.04	−2.41	0.138
Age	0.34	0.16	2.17	0.162
Gender (female)	5.83	2.60	2.24	0.154
Type of Control (active)	0.18	0.23	0.78	0.516
RoB assessment (low risk of bias)	0.08	0.19	0.47	0.654
**Outcome 6: Anxiety**				
Length of treatment (total duration in weeks)	NA	NA	NA	NA
Contact (daily contact)	NA	NA	NA	NA
Intensity (total hours per week)	NA	NA	NA	NA
Age	NA	NA	NA	NA
Gender (female)	NA	NA	NA	NA
Type of Control (active)	NA	NA	NA	NA
RoB assessment (low risk of bias)	NA	NA	NA	NA

RoB = risk of bias, SE = standard error, NA = not applicable.

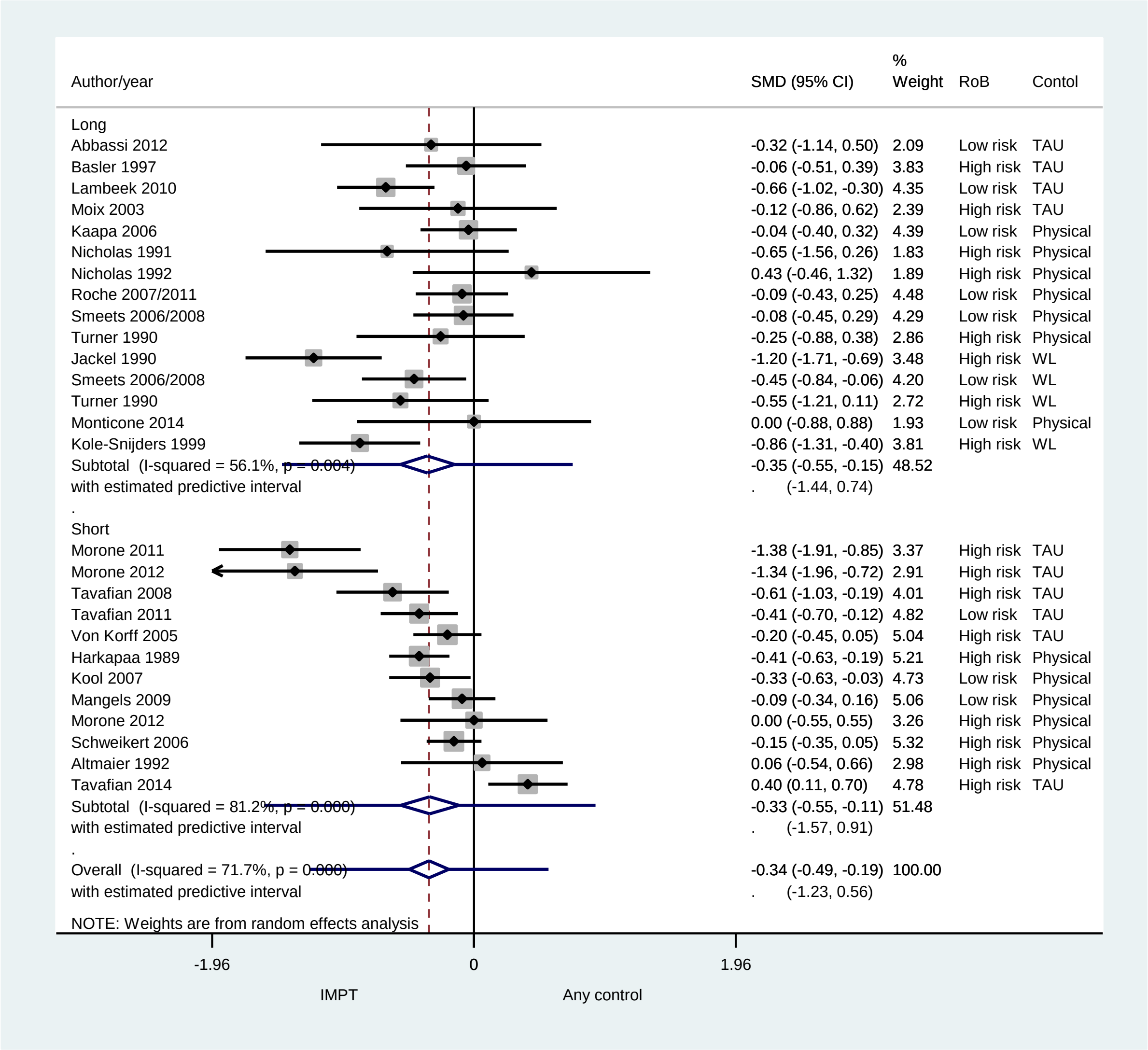

Figure S7. Sensitivity analysis by length for the pain outcome, after excluding the study of Moticone et al. (2013/2014).

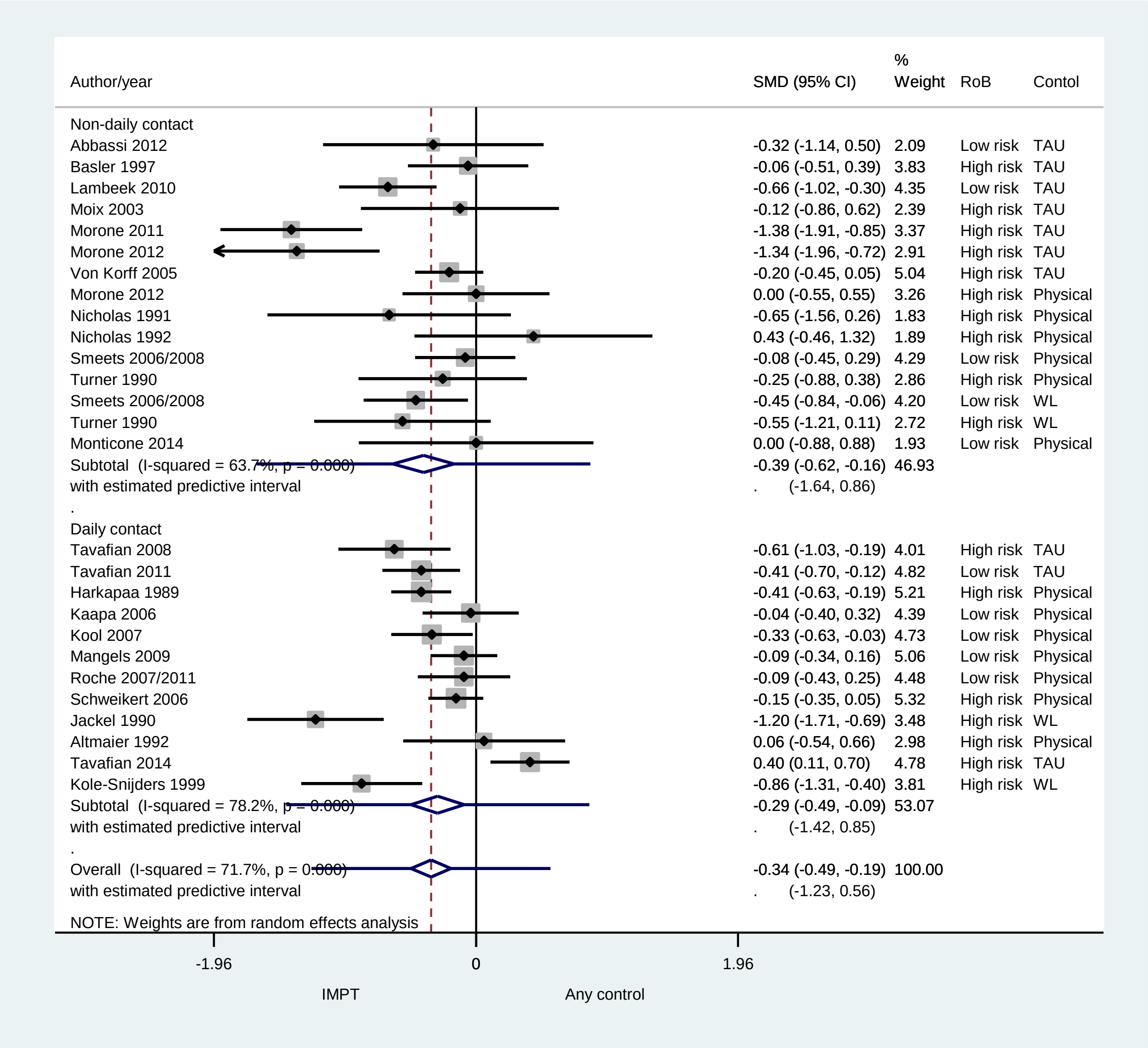

Figure S8. Sensitivity analysis by contact for the pain outcome, after excluding the study of Moticone et al. (2013/2014).

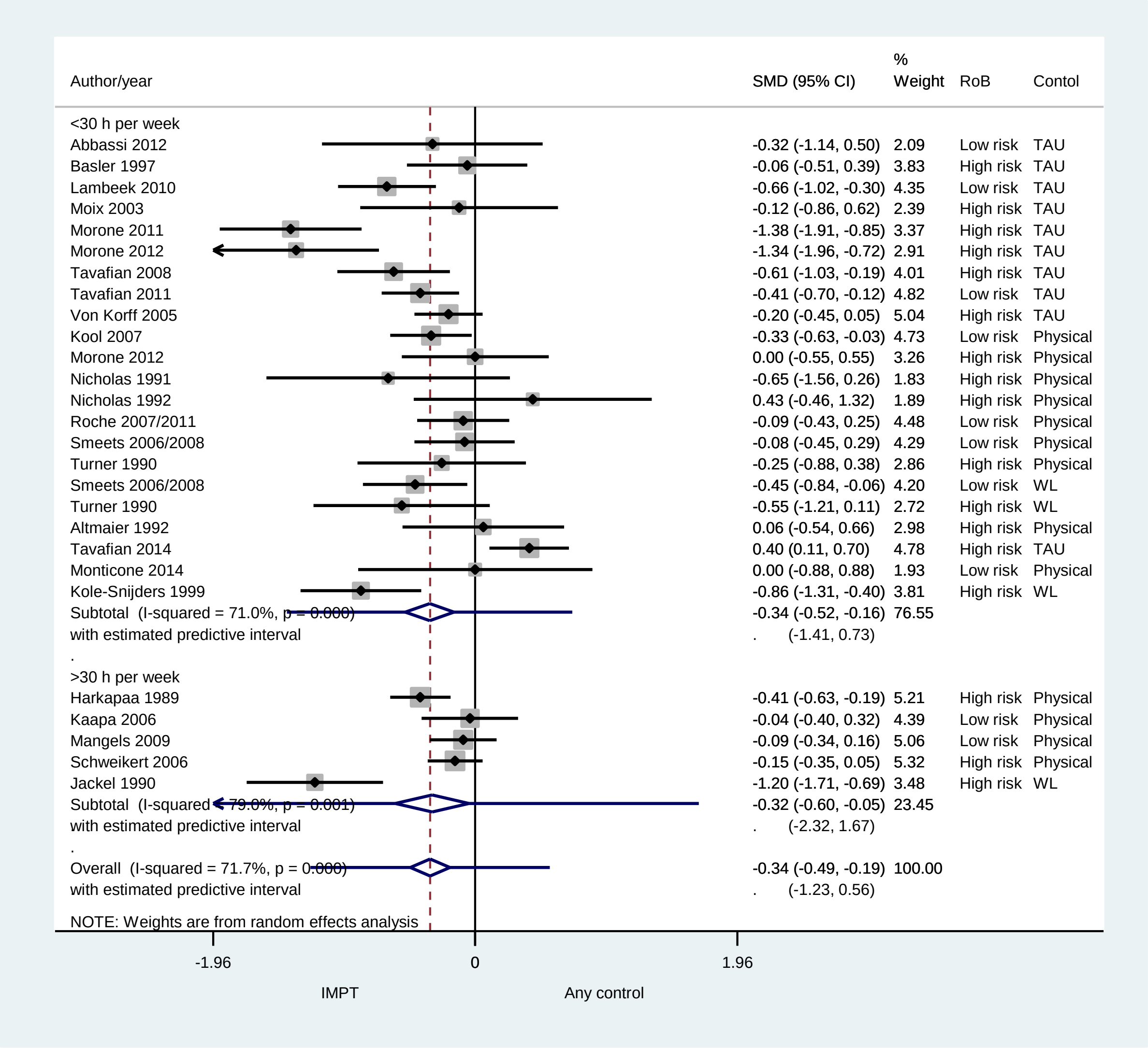

Figure S9. Sensitivity analysis by intensity for the pain outcome, after excluding the study of Moticone et al. (2013/2014).

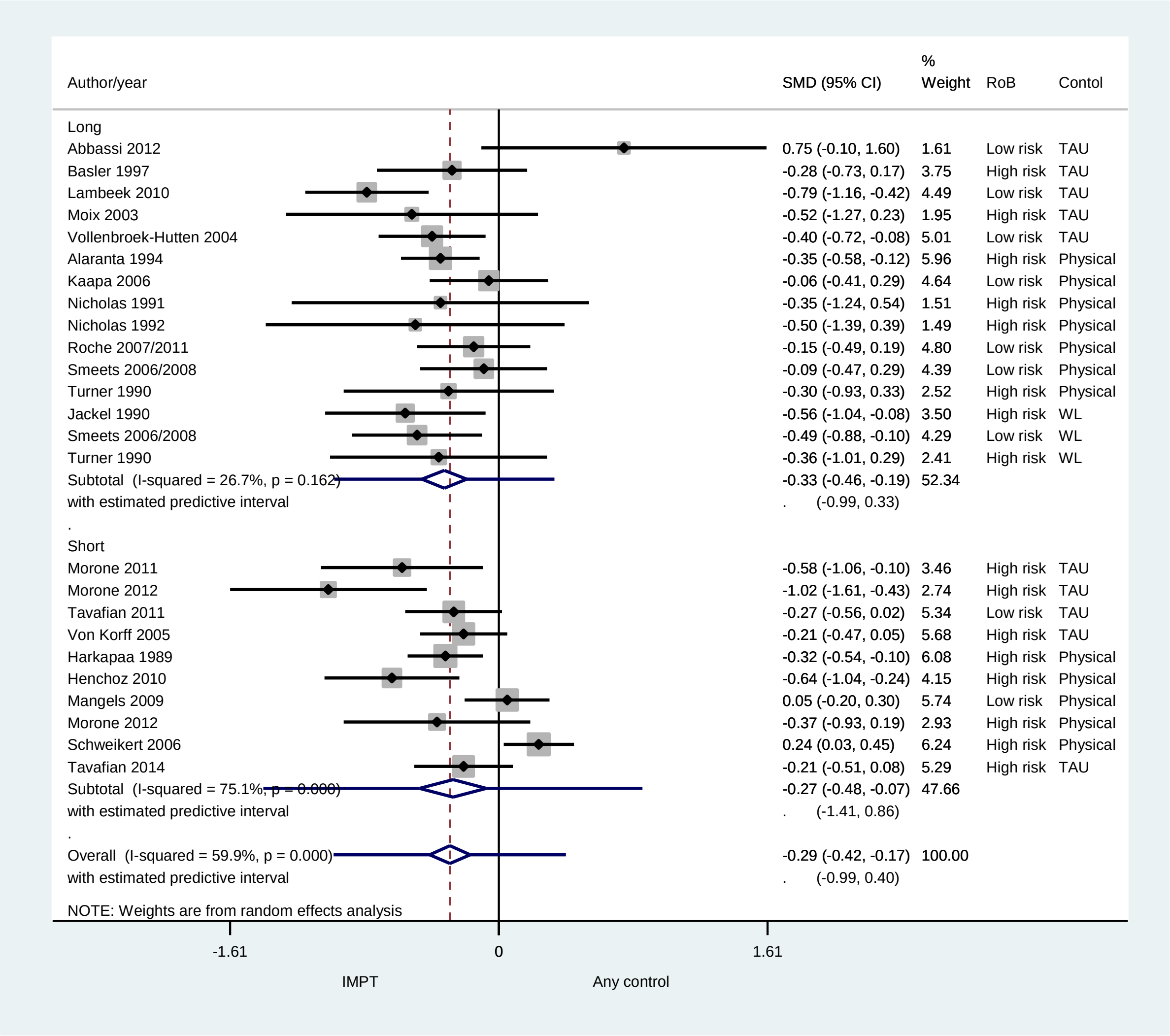

Figure S10. Sensitivity analysis by length for the disability outcome, after excluding the study of Moticone et al. (2013/2014).

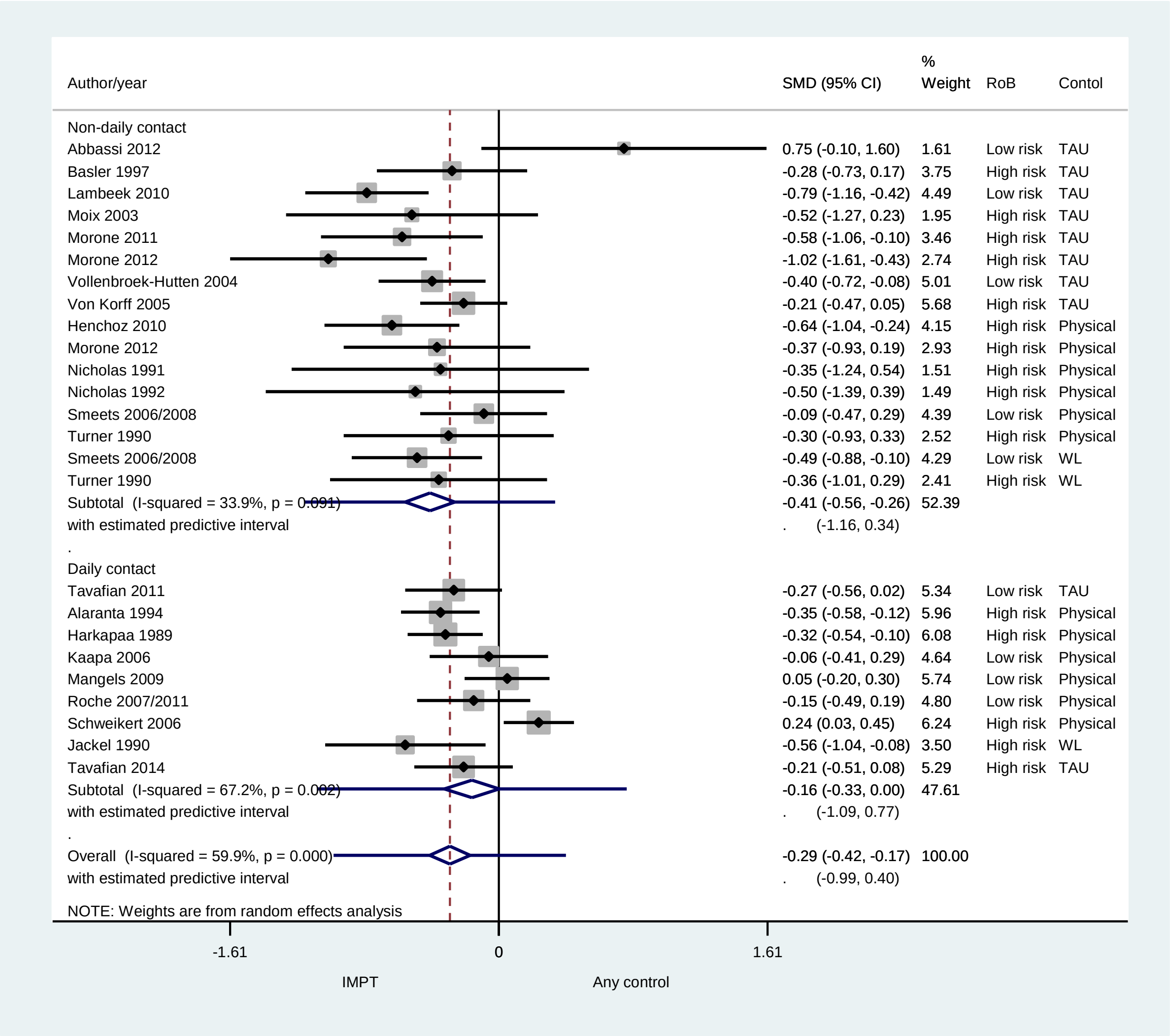

Figure S11. Sensitivity analysis by contact for the disability outcome, after excluding the study of Moticone et al. (2013/2014).

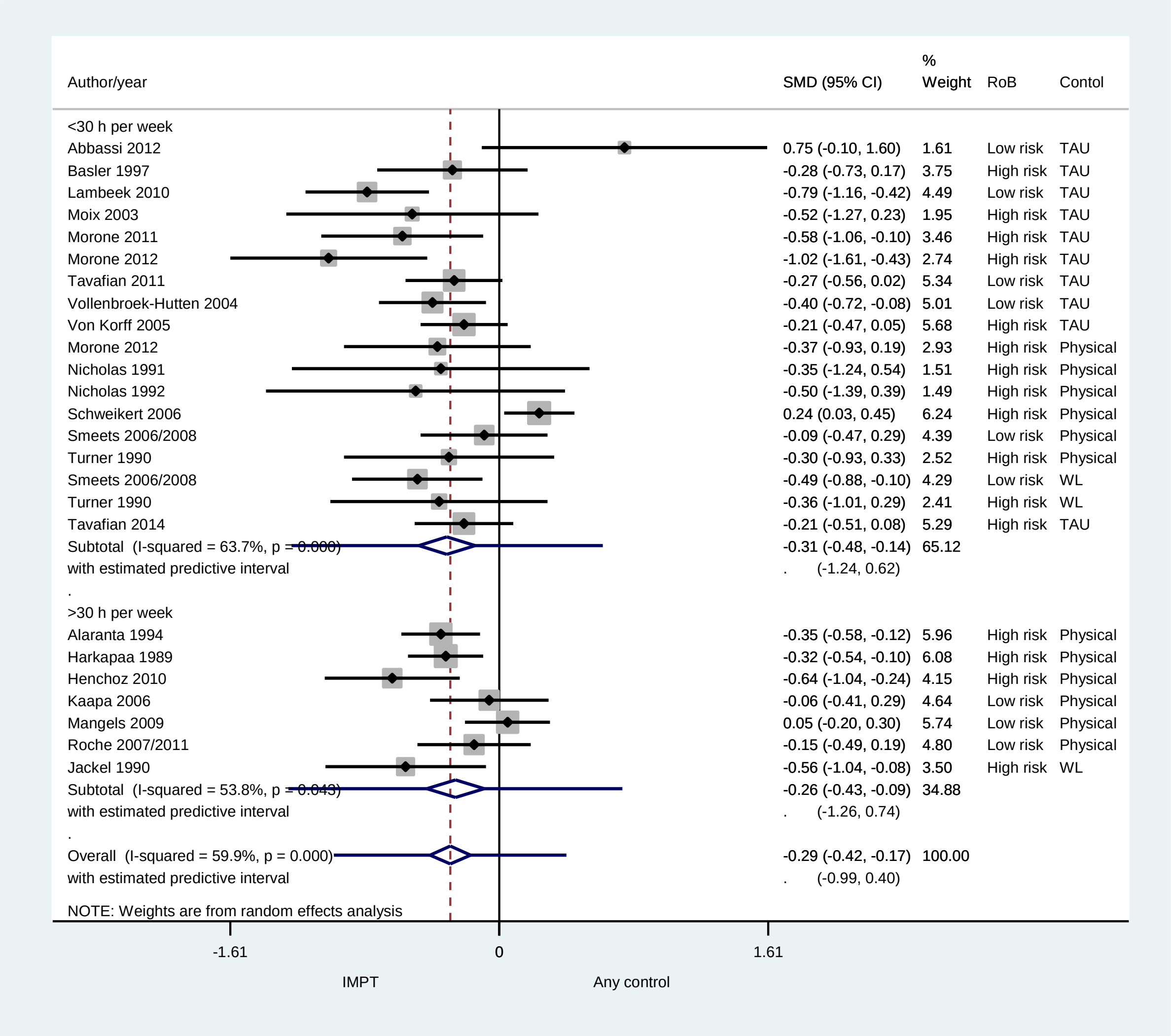

Figure S12. Sensitivity analysis by intensity for the disability outcome, after excluding the study of Moticone et al. (2013/2014).

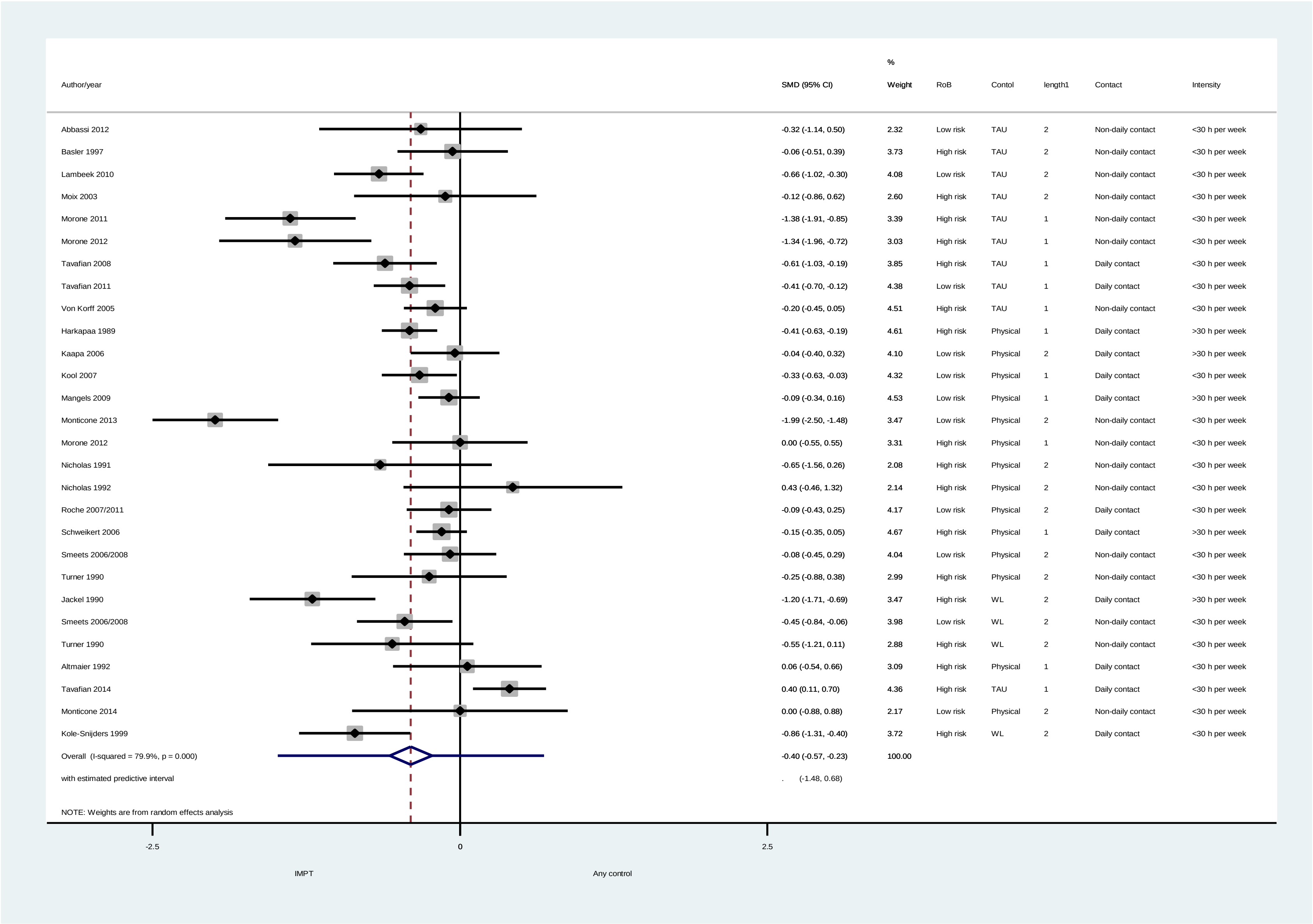

Figure S13. Forest plot for the pain outcome all studies included.

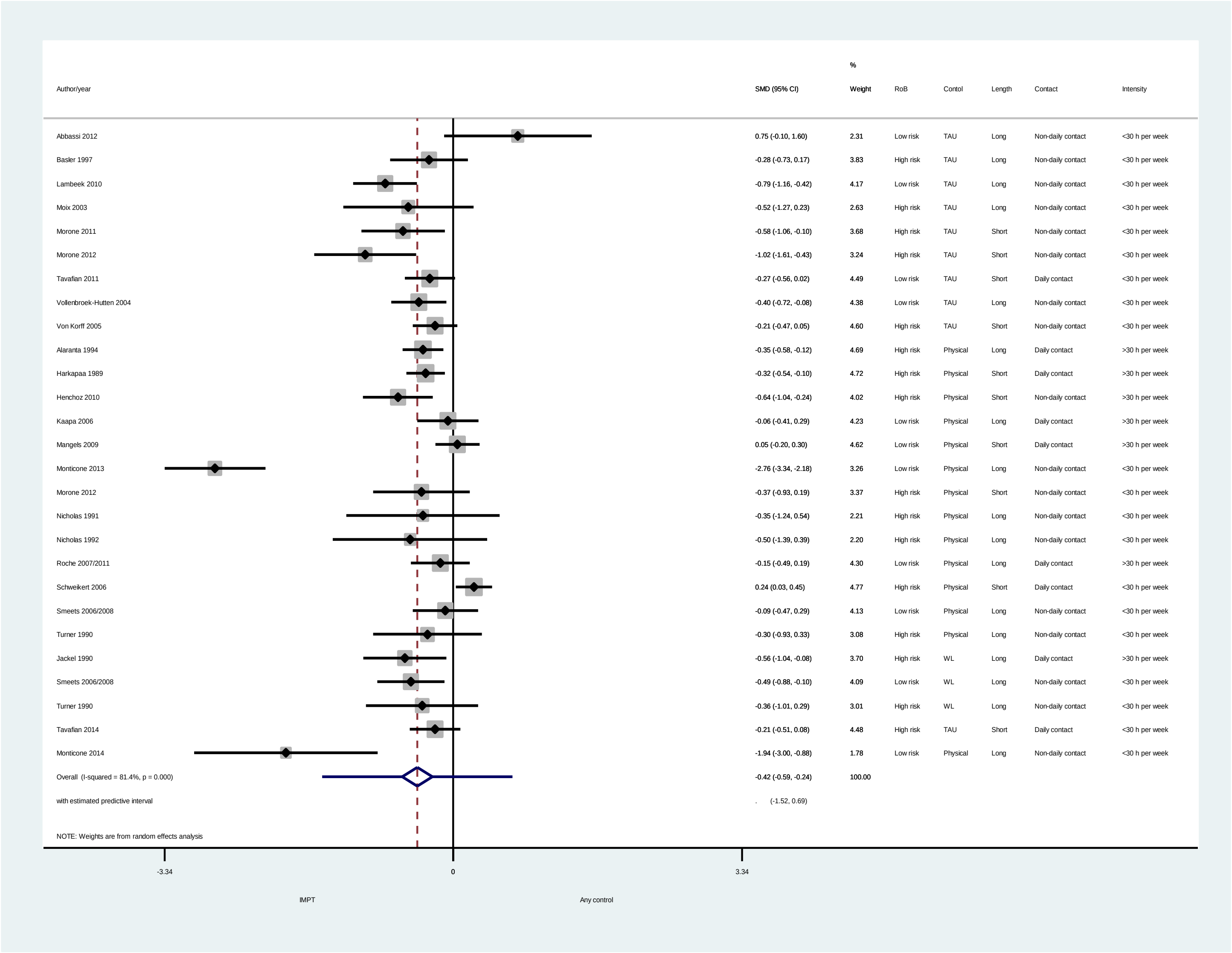

Figure S14. Forest plot for the disability outcome all studies included.

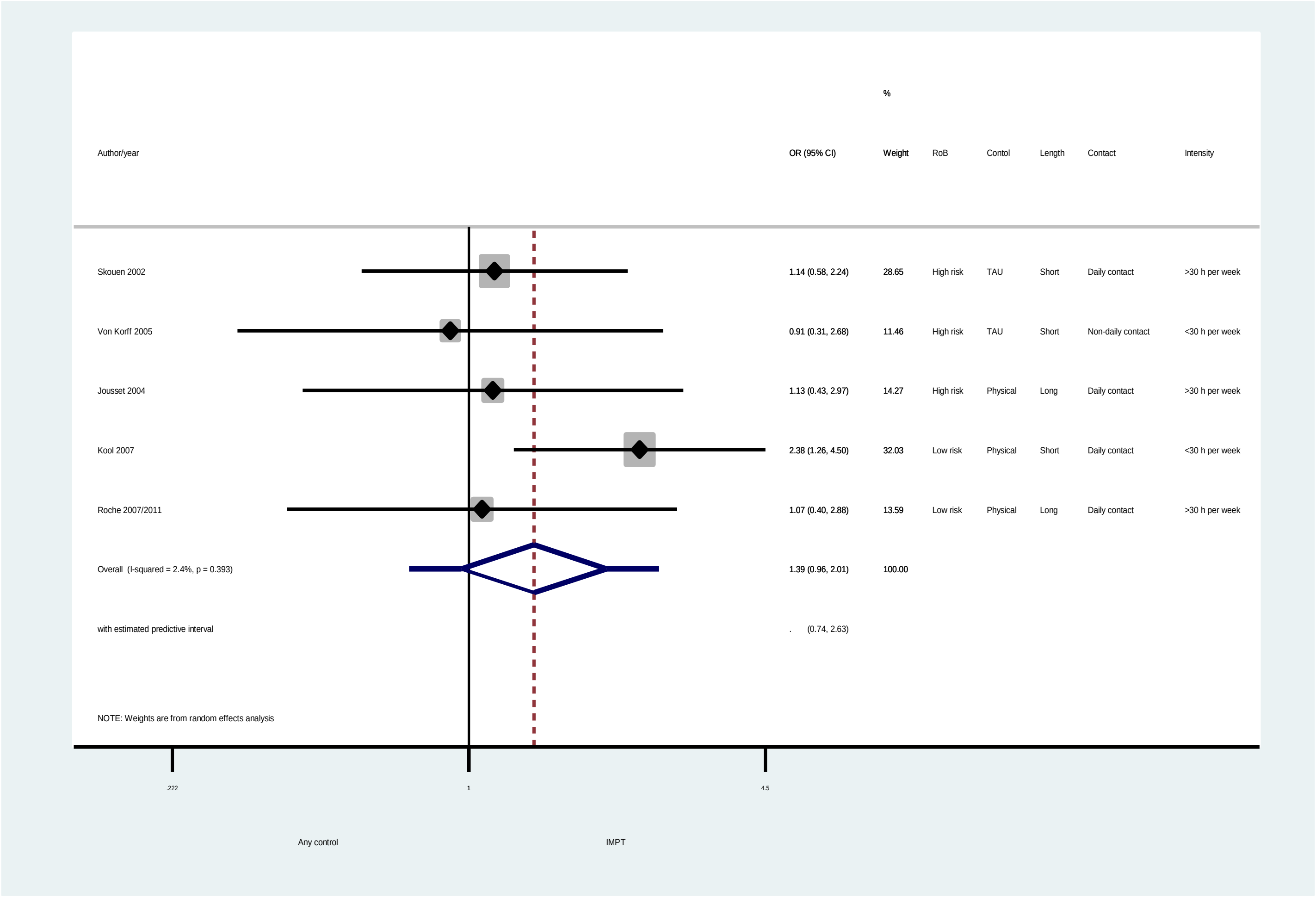

Figure S15. Forest plot for the return to work outcome all studies included.

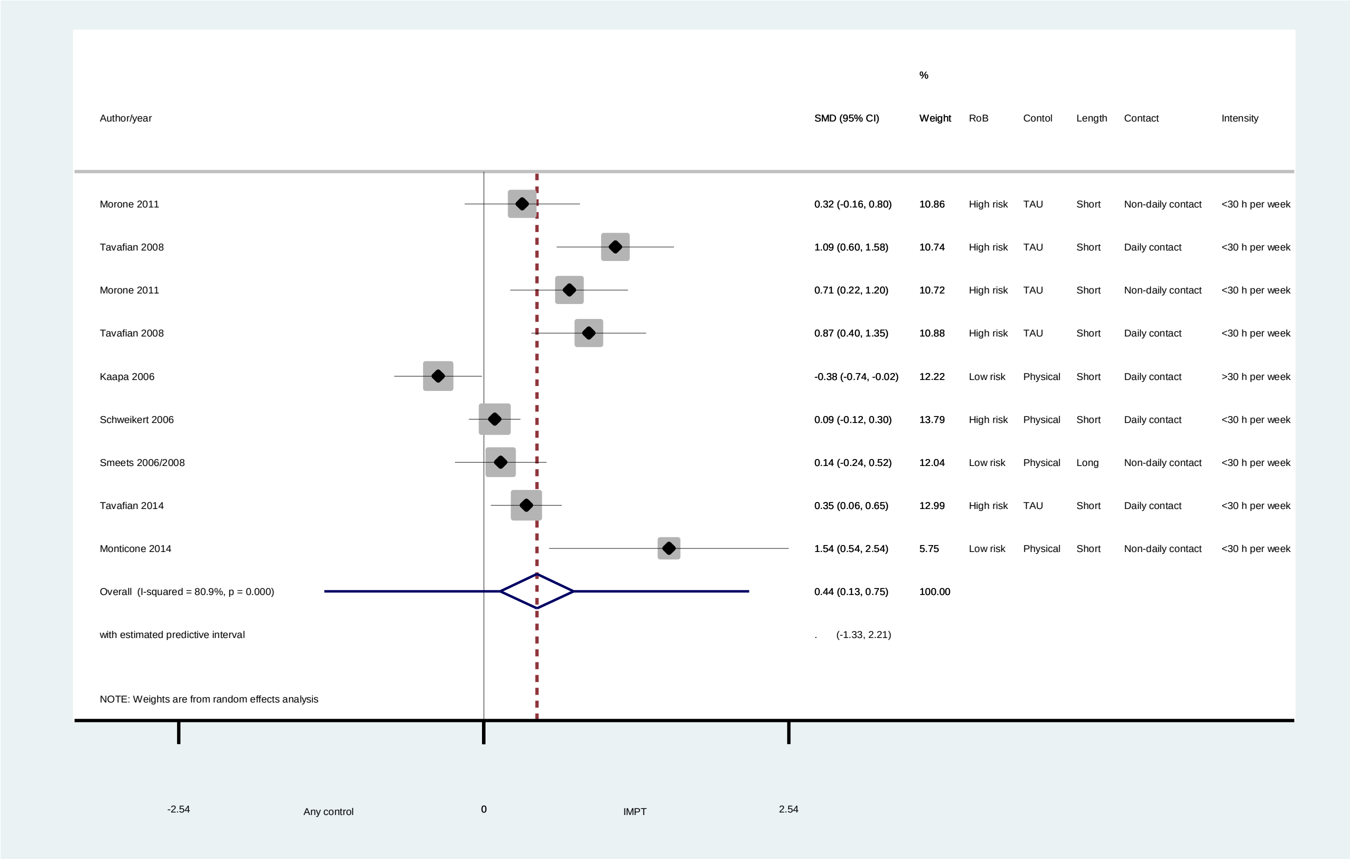

Figure S16. Forest plot for the quality of life outcome all studies included.

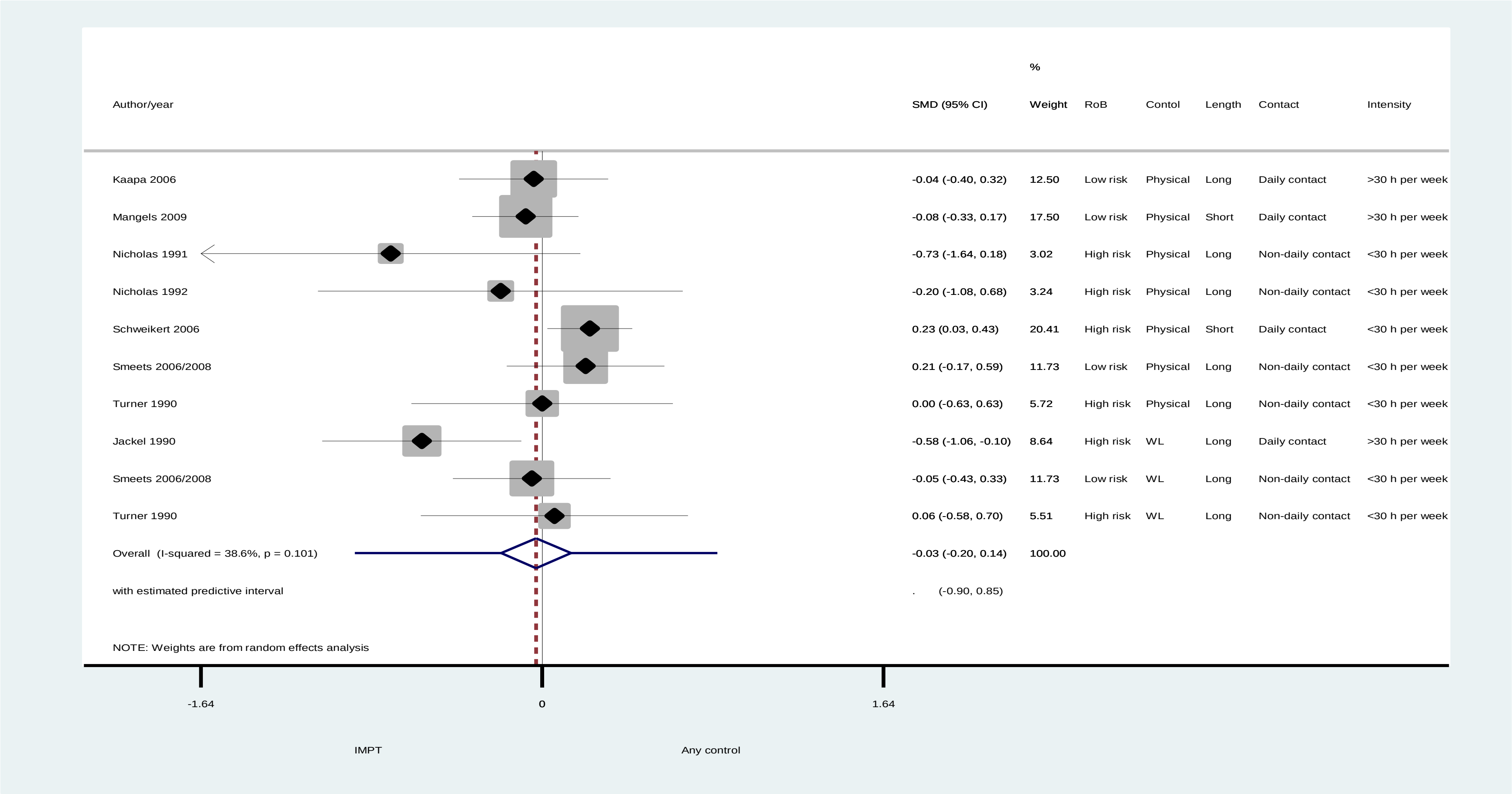

Figure S17. Forest plot for the depression outcome all studies included.

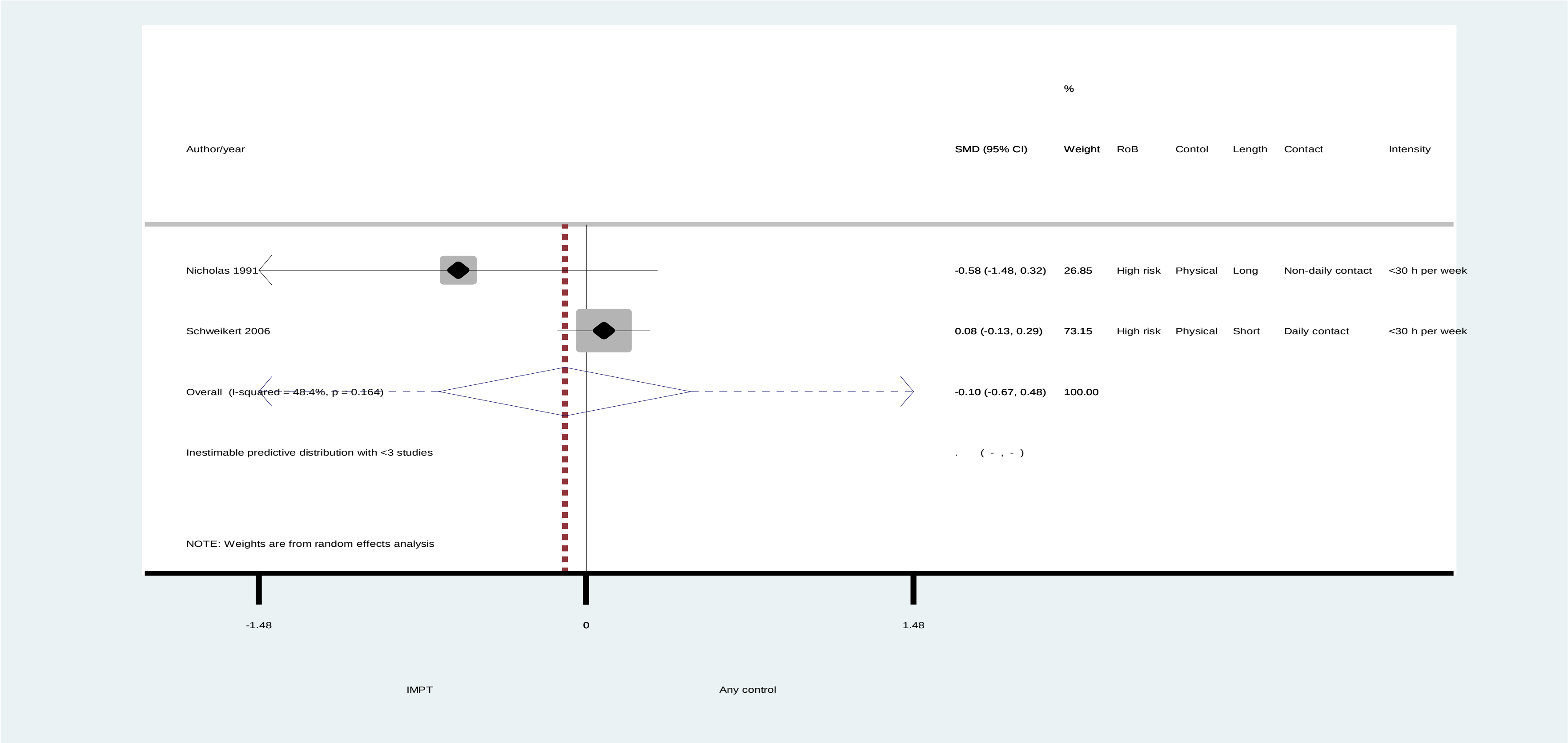

Figure S18. Forest plot for the anxiety outcome all studies included.

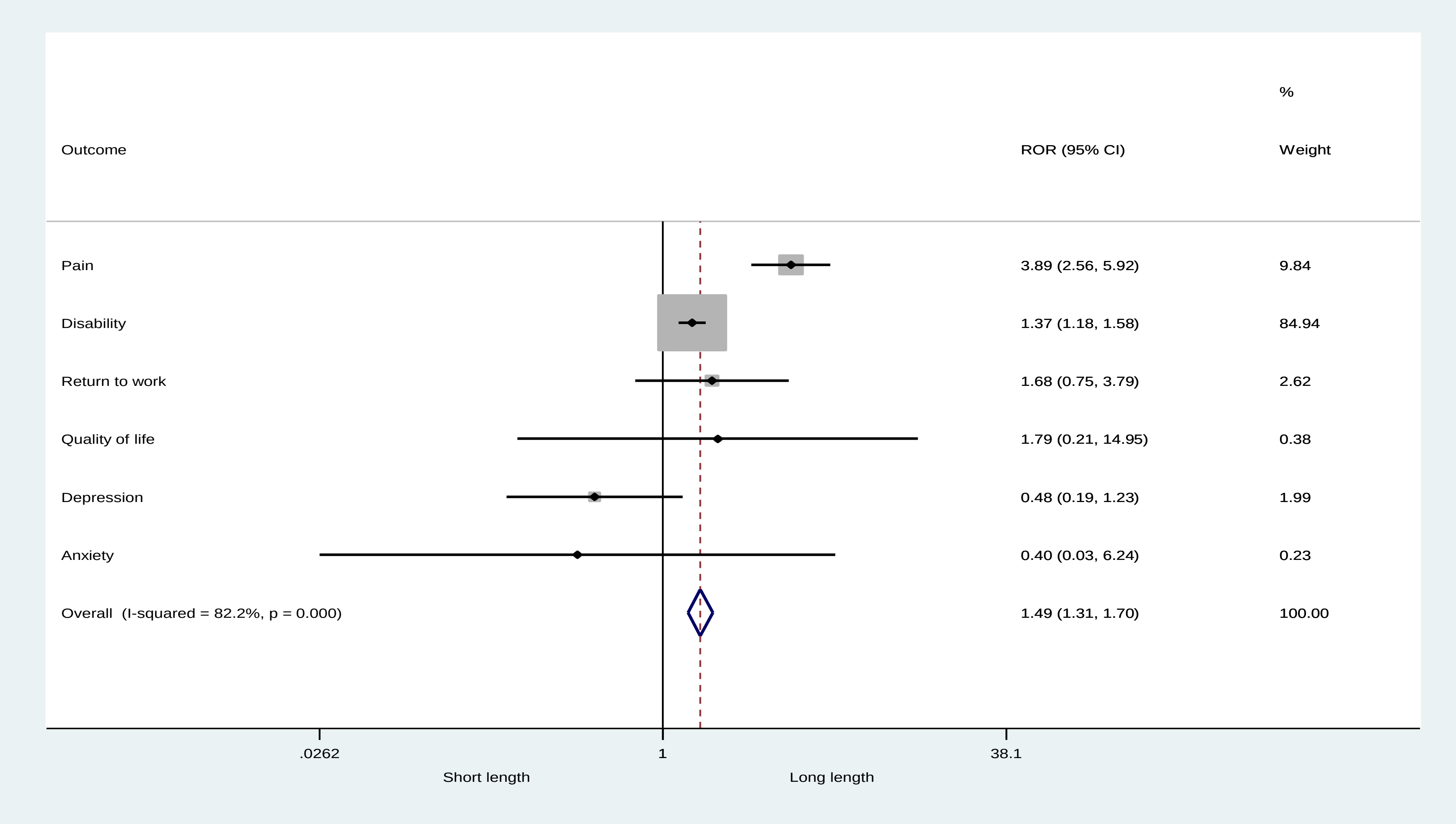

Figure S19. The relative odds ratios (RORs) and 95% confidence intervals (CIs) for each outcome at short term of a short-length treatment vs. long-length treatment. The RORs were calculated with a fixed-effect model. A ROR >1 favours long length; a ROR <1 favours short length.

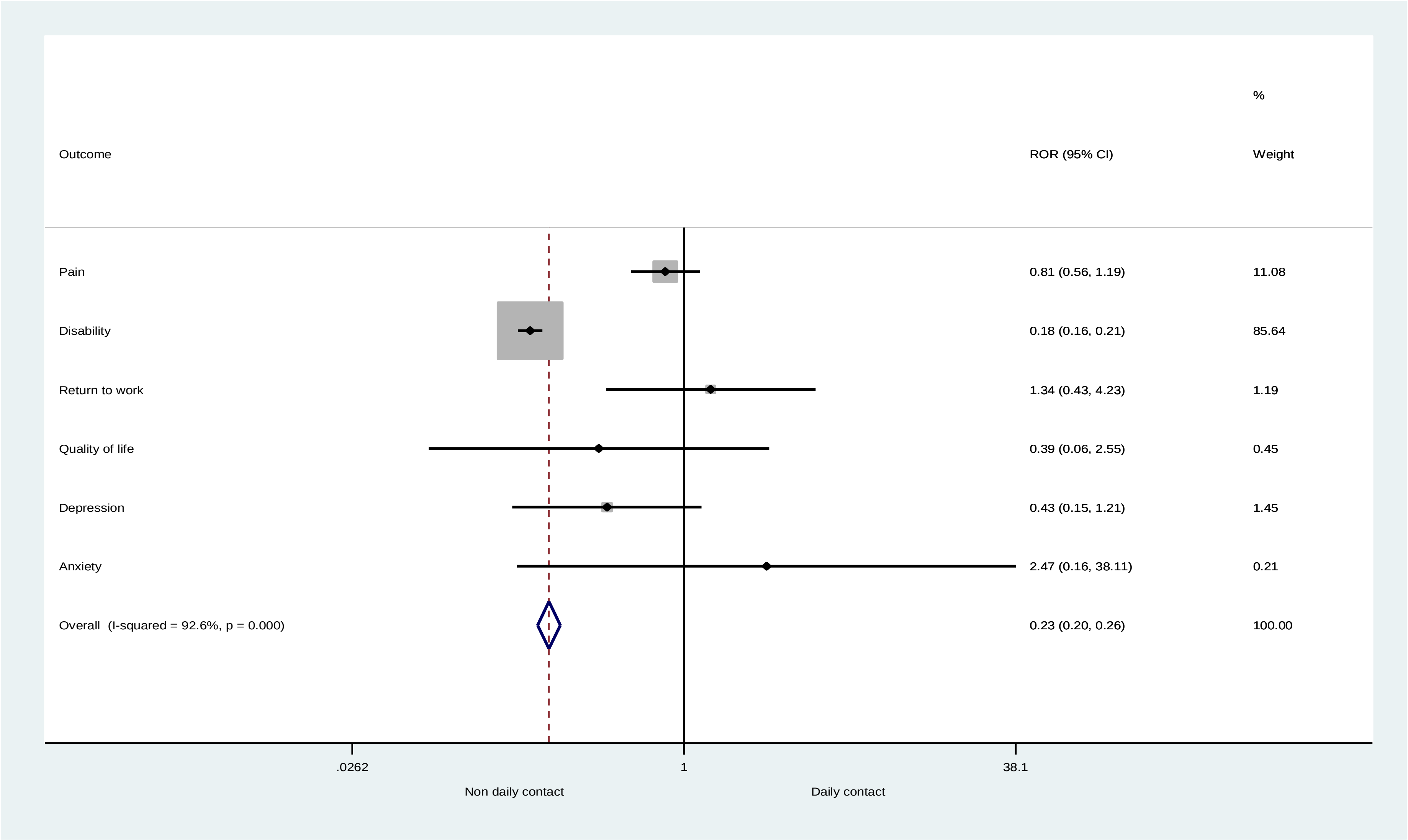

Figure S20. The relative odds ratios (RORs) and 95% confidence intervals (CIs) for each outcome at short term of a non-daily contact vs. daily contact. The RORs were calculated with a fixed-effect model. A ROR >1 favours daily contact; a ROR <1 favours non-daily contact.

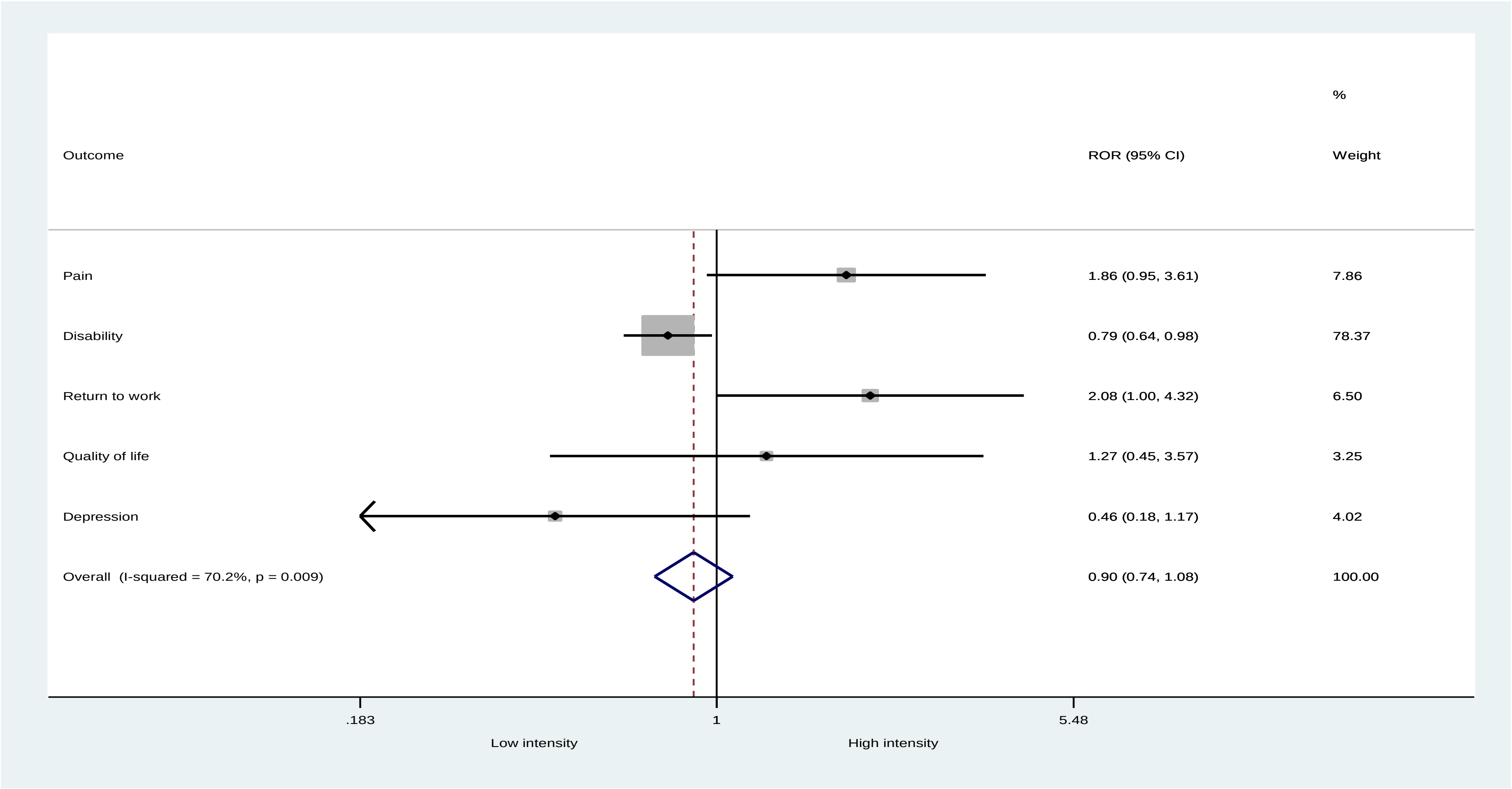

Figure S21. The relative odds ratios (RORs) and 95% confidence intervals (CIs) for each outcome at short term of a low intensity vs. high intensity. The RORs were calculated with a fixed-effect model. A ROR >1 favours high intensity (i.e., >30 h per week); a ROR <1 favours low intensity (i.e., <30 h per week).

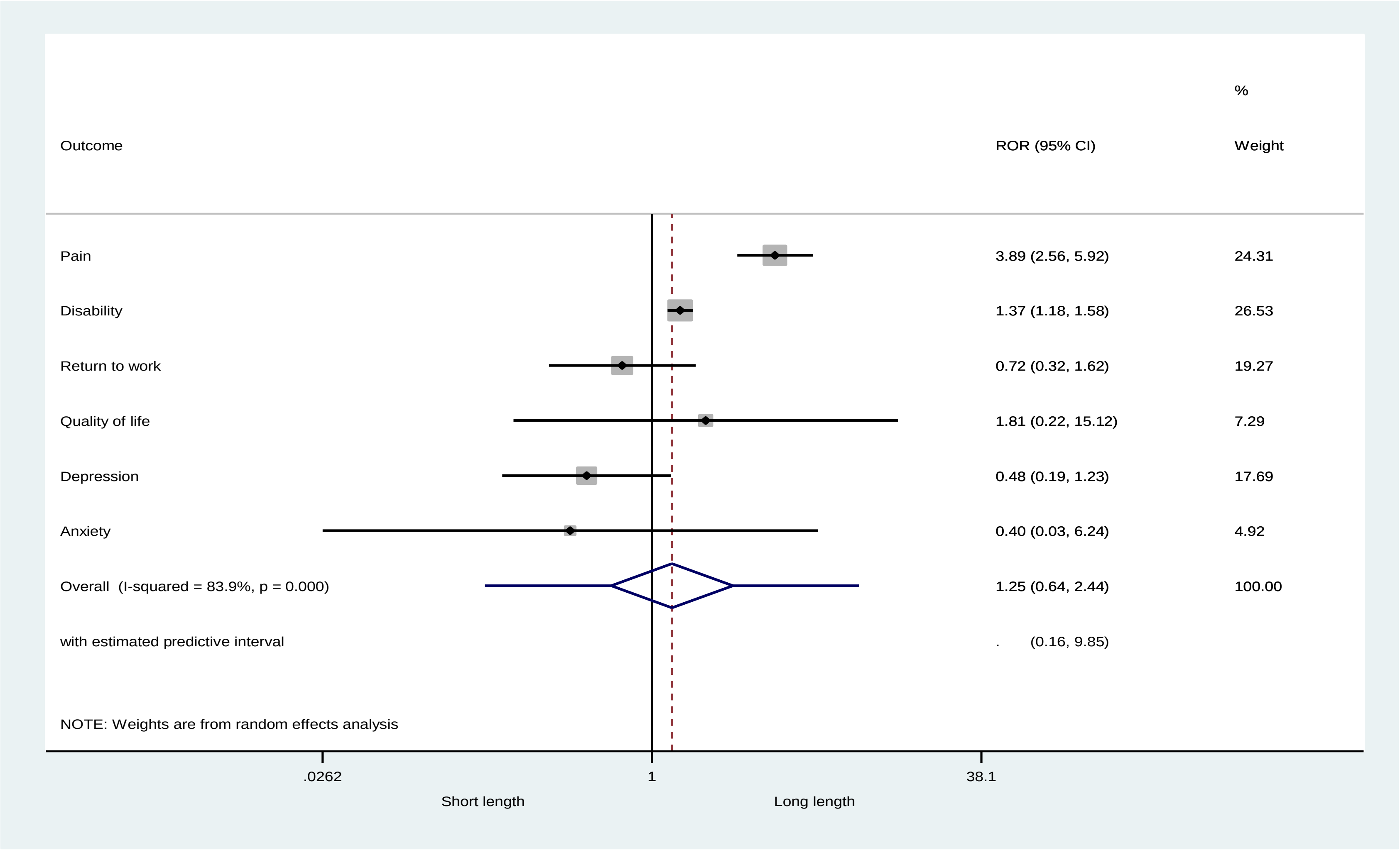

Figure S22. The relative odds ratios (RORs) and 95% confidence intervals (CIs) for each outcome at short term of a short-length treatment vs. long-length treatment, after excluding the study of Moticone et al. (2013/2014). The RORs were calculated with a random-effects model. A ROR >1 favours long length; a ROR <1 favours short length.

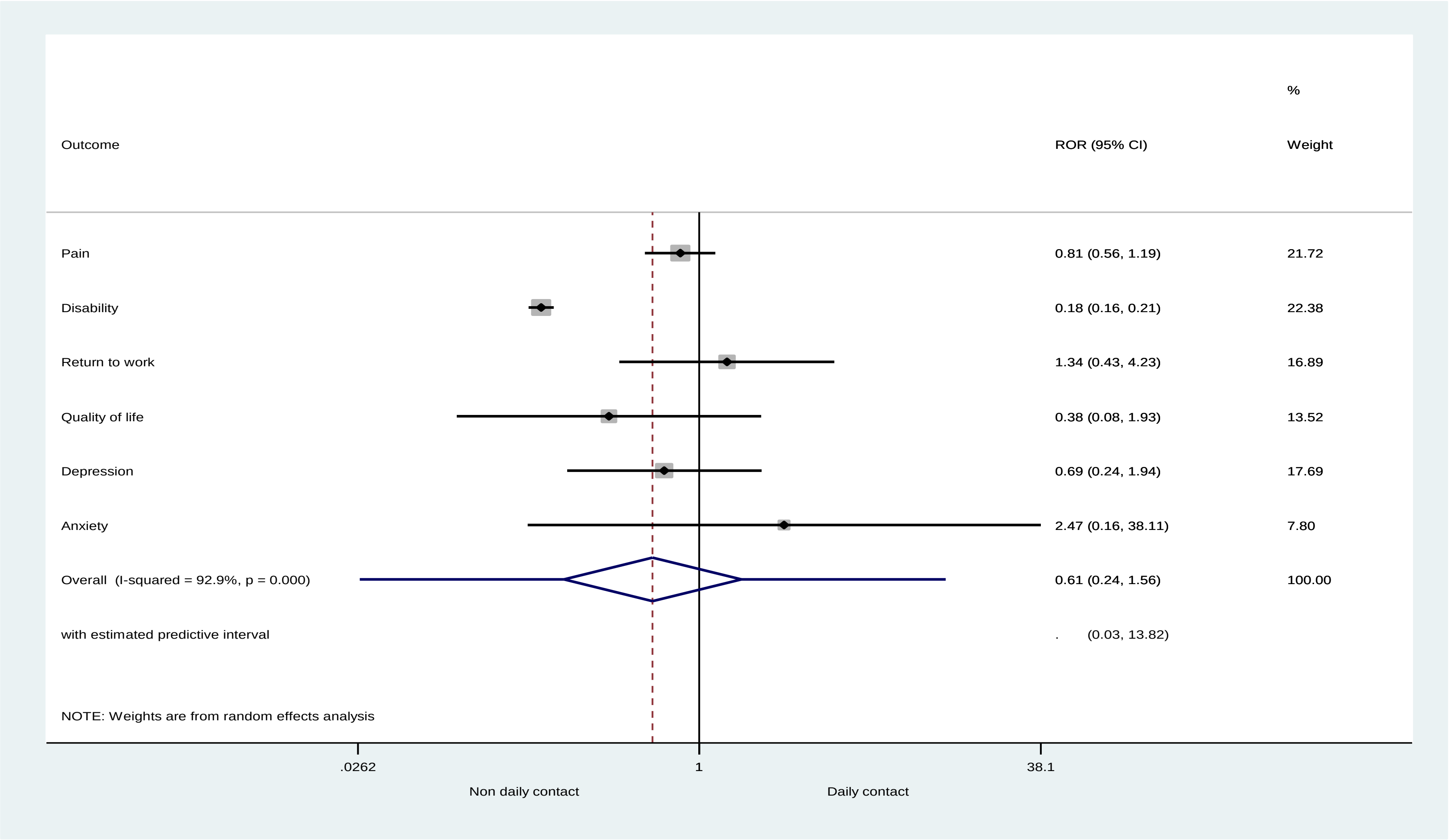

Figure S23. The relative odds ratios (RORs) and 95% confidence intervals (CIs) for each outcome at short term of a non-daily contact vs. daily contact, after excluding the study of Moticone et al. (2013/2014). The RORs were calculated with a random-effects model. A ROR >1 favours daily contact; a ROR <1 favours non-daily contact.

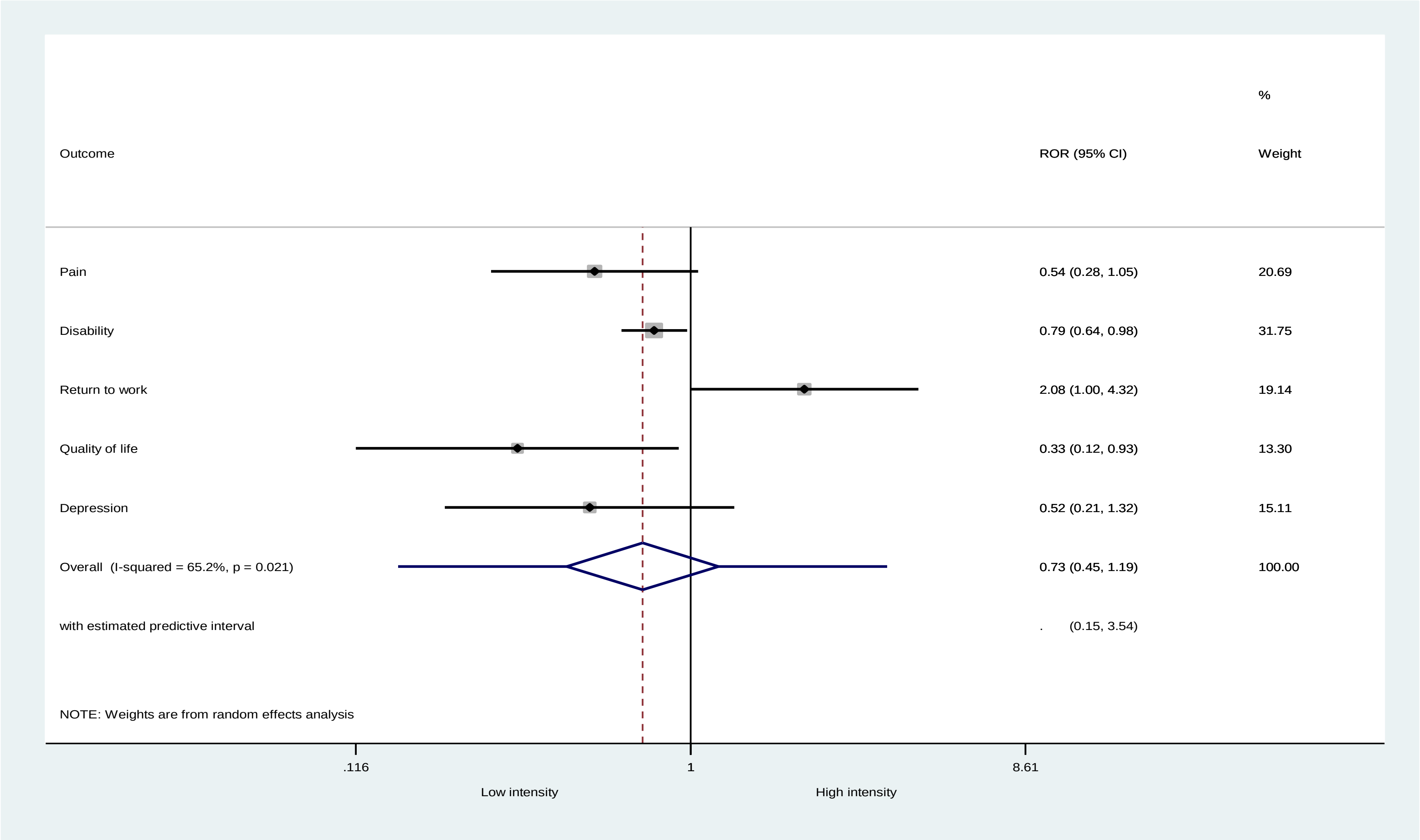

Figure S24. The relative odds ratios (RORs) and 95% confidence intervals (CIs) for each outcome at short term of a low intensity vs. high intensity, after excluding the study of Moticone et al. (2013/2014). The RORs were calculated with a random-effects model. A ROR >1 favours high intensity (i.e., >30 h per week); a ROR <1 favours low intensity (i.e., <30 h per week).

Box S2 e-References of included RCTs

1. Abbasi M, Dehghani M, Keefe FJ, Jafari H, Behtash H,Shams J. Spouse-assisted training in pain coping skills and the outcome of multidisciplinary pain management for chronic low back pain treatment: A 1-year randomized controlled trial. European Journal of Pain 2012;16(7):1033–43.
2. Alaranta H, Rytökoski U, Rissanen A, Talo S, Rönnemaa T, Puukka P, et al. Intensive physical and psychosocial training program for patients with chronic low back pain. A controlled clinical trial. Spine 1994;19:1339–49.
3. Altmaier EM, Lehmann TR, Russell DW, Weinstein JN, Kao CF. The effectiveness of psychological interventions for the rehabilitation of low back pain: a randomized controlled trial evaluation. Pain. 1992;49(3):329-35.
4. Basler H, Jakle C, Kroner-Herwig B. Incorporation of cognitive behavioral treatment into the medical care of chronic low back patients: A controlled randomized study in German pain treatment centers. Patient Education & Counseling 1997;31:113–24.
5. Bendix AF, Bendix T, Vaegter K, Lund C, Frolund L, Holm L. Multidisciplinary intensive treatment for chronic low back pain: a randomized, prospective study. Cleveland Clinic Journal of Medicine 1996;63:62–9.
6. Bendix AF, Bendix T, Ostenfeld S, Bush E, Andersen A. Active treatment programs for patients with chronic low back pain: a prospective, randomized, observer blinded study. European Spine Journal 1995;4:149–52.
7. Bendix T, Bendix A, Labriola M, Haestrup C, Ebbehoj N. Functional restoration versus outpatient physical training in chronic low back pain: a randomized comparative study. Spine 2000;25(19):2494–500.
8. Coole C, Drummond A, Watson P. Individual work support for employed patients with low back pain: a randomized controlled pilot trial. Clinical Rehabilitation 2013;27(1):40–50.
9. Corey DT, Koepfler LE, Etlin D, Day HI. A limited functional restoration program for injured workers: A randomized trial. J Occup Rehabil. 1996 Dec;6(4):239-49.
10. Fairbank J, Frost H, Wilson-MacDonald J, Yu L-M, Barker K, Collins R. Randomised controlled trial to compare surgical stabilisation of the lumbar spine with an intensive rehabilitation programme for patients with chronic low back pain: the MRC spine stabilisation trial. BMJ 2005;330(7502):1233–8.
11. Harkapaa K, Jarvikoski A, Mellin G, Hurri H. Pain, disability, compliance, and reported treatment benefits three months after treatment. Scandinavian Journal of Rehabilitation Medicine 1989;21:81–9.
12. Hellum C, Johnsen LG, Storheim K, Nygaard O P, Brox JI, Rossvoll I, et al. Surgery with disc prosthesis versus rehabilitation in patients with low back pain and degenerative disc: two year follow-up of randomised study.BMJ 2011;342:d2786.
13. Henchoz Y, de Goumoens P, So AK, Paillex R. Functional multidisciplinary rehabilitation versus outpatient physiotherapy for non specific low back pain: randomized controlled trial. Swiss Medical Weekly 2010;140(w13133):1–7.
14. Jackel WH, Cziske R, Gerdes N, Jacobi E. Assessment of the effectiveness of inpatient rehabilitation measures in patients with chronic low back pain: a prospective, randomized, controlled study [German]. Rehabilitation 1990;29:129–33.

15. Jousset N, Fanello S, Bontoux L, Dubus V, Billabert C, Vielle B. Effects of functional restoration versus 3 hours per week physical therapy: a randomized controlled study. Spine 2004;29(5):487–94.
16. Kaapa EH, Frantsi K, Sarna S, Malmivaara A. Multidisciplinary group rehabilitation versus individual physiotherapy for chronic nonspecific low back pain: a randomized trial. Spine 2006;31(4):371–6.
17. Kole-Snijders AMJ, Vlaeyen JWS, Goossens MEJB, Rutten-Van Molken MPMH, Heuts PHTG, Van Breukelen G, Van Eek H. Chronic low-back pain: what does cognitive coping skills training add to operant behavioral treatment? Results of a randomized clinical trial. Journal of Consulting and Clinical Psychology 1999;67(6):931–44.
18. Kool J, Bachmann S, Oesch P, Knuesel O, Ambergen T, de Bie R. Function-centered rehabilitation increases work days in patients with nonacute nonspecific low back pain: 1-year results from a randomized controlled trial. Archives of Physical Medicine and Rehabilitation 2007;88(9):1089–94.
19. Lambeek LC, vanMechelen W, Knol DL, Loisel P, Anema JR. Randomised controlled trial of integrated care to reduce disability from chronic low back pain in working and private life. BMJ 2010;340(7749):750.
20. Leeuw M, Goossens MEJB, van Breukelen GJP, de Jong JR, Heuts PHTG, Smeets RJEM, et al. Exposure in vivo versus operant graded activity in chronic low back pain patients: results of a randomized controlled trial. Pain 2008;138: 192–207.
21. Linton SJ, Boersma K, Jansson M, Svärd L, Botvalde M. The effects of cognitive-behavioral and physical therapy preventive interventions on pain-related sick leave: a randomized controlled trial. Clinical Journal of Pain 2005; 21(2):109–19.
22. Lukinmaa A. Low back pain as a biopsychosocial problem.A controlled clinical trial and a cost effectiveness analysis [Finnish]. Kansanelakelaitoksen Julkaisuja 1989;ML:1–90.
23. Mangels M, Schwarz S, Worringen U, Holme M, Rief W. Evaluation of a behavioral-medical inpatient rehabilitation treatment including booster sessions: a randomized controlled study. Clinical Journal of Pain 2009;25(5): 356–64.
24. Meng K, Seekatz B, Roband H, Worringen U, Vogel H, Faller H. Intermediate and long-term effects of a standardized back school for inpatient orthopedic rehabilitation on illness knowledge and self-management behaviors: a randomized controlled trial. Clinical Journal of Pain 2011;27(3):248–57.
25. Mitchell RI, Carmen GM. The functional restoration approach to the treatment of chronic pain in patients with soft tissue and back injuries. Spine 1994;19:633–42.
26. Moix J, Canellas M, Osorio C, Bel X, Girvent F, Martos A. Efficacy of an interdisciplinary educational program in patients with chronic back pain [Spanish]. Dolor 2003;18(3):149–57.
27. Monticone M, Ferrante S, Rocca B, Baiardi P, Dal Farra F, Foti C. Effect of a long-lasting multidisciplinary program on disability and fear-avoidance behaviors in patients with chronic low back pain: results of a randomized controlled trial. Clinical Journal of Pain 2013;29(11):929–38.
28. Monticone M, Ambrosini E, Rocca B, Magni S, Brivio F, Ferrante S. A multidisciplinary rehabilitation programme improves disability, kinesiophobia and walking ability in subjects with chronic low back pain: results of a randomised controlled pilot study. Eur Spine J. 2014;23(10):2105-13.

29. Morone G, Paolucci T, Alcuri MR, Vulpiani MC, Matano A, Bureca I. Quality of life improved by multidisciplinary back school program in patents with chronic non-specific low back pain: a single blind randomized controlled trial. Europan Journal of Physical and Rehabilitation Medicine 2011;47(4):533–41.
30. Morone G, IosaM, Paolucci T, Fusco A, Alcuri R, Spadini E. Efficacy of perceptive rehabilitation in the treatment of chronic nonspecific low back pain through a new tool: a randomized clinical study. Clinical Rehabilitation 2012;26 (4):339–50.
31. Nicholas MK, Wilson PH, Goyen J. Operant-behavioural and cognitive behavioural treatment for chronic low back pain. Behavioural Research and Therapy 1991;29:225–38.
32. Nicholas MK, Wilson PH, Goyen J. Comparison of cognitive behavioral group treatment and an alternative non-psychological treatment for chronic low back pain. Pain 1992;48:339–47.
33. Roche G, Ponthieux A, Parot-Shinkel E, Jousset N, Bontoux L, Dubus V. Comparison of a functional restoration program with active individual physical therapy for patients with chronic low back pain: a randomized controlled trial. Archives of Physical Medicine and Rehabilitation 2007;88(10):1229–35
34. Smeets RJEM, Vlaeyen JWS, Hidding A, Kester ADM,van der Heijden GJMG, van Geel ACM, Knottnerus JA. Active rehabilitation for chronic low back pain :Cognitive-behavioral, physical, or both? First direct posttreatmentresults from a randomized controlled trial. BMC Musculoskeletal Disorders 2006;7:5.
35. Skouen JS, Grasdal AL, Haldorsen EM, Ursin H. Relative cost-effectiveness of extensive and light multidisciplinary treatment programs versus treatment as usual for patients with chronic low back pain on long-term sick leave: randomized controlled study. Spine 2002;27(9):901–9.
36. Schweikert B, Jacobi E, Seitz R, Cziske R, Ehlert A, Knab J. Effectiveness and cost-effectiveness of adding a cognitive behavioral treatment to the rehabilitation of chronic low back pain. Journal of Rheumatology 2006;33(12):2519–26.
37. Strand LI, Ljunggren AE, Haldorsen EMH, Espehaug B. The impact of physical function and pain on work status at 1-year follow-up in patients with back pain. Spine 2001;26(7):800–8.
38. Streibelt M, Thren K, Muller-Fahrnow W. Effects of FCE-based multidisciplinary rehabilitation in patients with chronic musculoskeletal disorders - results of a randomized controlled trial [German]. Physikalische Medizin Rehabilitationsmedizin Kurortmedizin 2009;19(1):34–41.
39. Tavafian SS, Jamshidi AR, Montazeri A. A randomizedstudy of back school in women with chronic low back pain: quality of life at three, six, and twelve months follow-up. Spine 2008;33(15):1617–21.
40. Tavafian SS, Jamshidi AR, Mohammad K. Treatmentof chronic low back pain: a randomized clinical trial comparing multidisciplinary group-based rehabilitation program and oral drug treatment with oral drug treatment alone. Clinical Journal of Pain 2011;27(9):811–8.
41. Tavafian SS, Jamshidi AR, Shay B. Treatment of low back pain: First extended follow up of an original trial (NCT00600197) comparing a multidisciplinary group-based rehabilitation program with oral drug treatment alone up to 24 months. Int J Rheum Dis 2017;20(12):1902-1909.

42. Tavafian SS, Jamshidi AR, Mohammad K. Treatment of low back pain: randomized clinical trial comparing a multidisciplinary group-based rehabilitation program with oral drug treatment up to 12 months. Int J Rheum Dis 2014;17(2):159-64.
43. Tavafian SS, Jamshidi AR, Mohammad K. Treatment of low back pain: Second extended follow up of an original trial (NCT00600197) comparing a multidisciplinary group-based rehabilitation program with oral drug treatment alone up to 30 months. Int J Rheum Dis. 2017;20(12):1910-1916.
44. Turner JA, Clancy S, McQuade KJ, Cardenas DD.Effectiveness of behavioral therapy for chronic low backpain: a component analysis. Journal of Consulting and Clinical Psychology 1990;58(5):573–9.
45. Van den Hout JHC, Vlaeyen JWS, Heuts PH, Zijlema JH,Wijnen JA. Secondary prevention of work-related disabilityin nonspecific low back pain: does problem-solving therapy help? A randomized clinical trial. Clinical Journal of Pain 2003;19(2):87–96.
46. Vollenbroek-Hutten MM, Hermens HJ, Wever D, Gorter M, Rinket J, Ijzerman MJ. Differences in outcome of a multidisciplinary treatment between subgroups of chronic low back pain patients defined using two multiaxial assessment instruments: the multidimensional pain inventory and lumbar dynamometry. Clin Rehabil. 2004;18(5):566-79.
47. Von Korff M, Balderson BH, Saunders K, Miglioretti DL, Lin EH, Berry S. A trial of an activating intervention for chronic back pain in primary care and physical therapy settings. Pain 2005;113(3):323–30.

Table S3. Checklist summarising compliance with PRISMA guidelines.

Section/topic	#	Checklist item	Reported on page #
**TITLE**			
Title	1	Identify the report as a systematic review, meta-analysis, or both.	1
**ABSTRACT**			
Structured summary	2	Provide a structured summary including the following: background; objectives; data sources; study eligibility criteria, participants, and interventions; study appraisal and synthesis methods; results; limitations; conclusions and implications of key findings; and systematic review registration number.	2
**INTRODUCTION**			
Rationale	3	Describe the rationale for the review in the context of what is already known.	3-4
Objectives	4	Provide an explicit statement of questions being addressed with reference to participants, interventions, comparisons, outcomes, and study design (PICOS).	4
**METHODS**			
Protocol and registration	5	Indicate if a review protocol exists, if and where it can be accessed (e.g., Web address), and, if available, provide registration information including registration number.	NA
Eligibility criteria	6	Specify study characteristics (e.g., PICOS and length of follow-up) and report characteristics (e.g., years considered, language, and publication status) used as criteria for eligibility and giving rationale.	4
Information sources	7	Describe all information sources (e.g., databases with dates of coverage and contact with study authors to identify additional studies) in the search and date last searched.	4
Search	8	Present full electronic search strategy for at least one database, including any limits used, such that it could be repeated.	Box S1
Study selection	9	State the process for selecting studies (i.e., screening, eligibility, included in systematic review, and, if applicable, included in the meta-analysis).	4-5
Data collection process	10	Describe method of data extraction from reports (e.g., piloted forms, independently, in duplicate) and any processes for obtaining and confirming data from investigators.	5

Data items	11	List and define all variables for which data were sought (e.g., PICOS and funding sources) and any assumptions and simplifications made.	5
Risk of bias in individual studies	12	Describe methods used for assessing risk of bias of individual studies (including specification of whether this was done at the study or outcome level) and how this information is to be used in any data synthesis.	5
Summary measures	13	State the principal summary measures (e.g., risk ratio and difference in means).	6
Synthesis of results	14	Describe the methods of handling data and combining results of studies, if done, including measures of consistency (e.g., I^2) for each meta-analysis.	6-7
**Section/topic**	**#**	**Checklist item**	**Reported on page #**
Risk of bias across studies	15	Specify any assessment of risk of bias that may affect the cumulative evidence (e.g., publication bias and selective reporting within studies).	6
Additional analyses	16	Describe methods of additional analyses (e.g., sensitivity or subgroup analyses, meta-regression), if done, indicating which were pre-specified.	7
**RESULTS**			
Study selection	17	Give numbers of studies screened, assessed for eligibility, and included in the review, with reasons for exclusions at each stage, ideally with a flow diagram.	7
Study characteristics	18	For each study, present characteristics for which data were extracted (e.g., study size, PICOS, and follow-up period) and provide the citations.	7
Risk of bias within studies	19	Present data on risk of bias of each study and, if available, any outcome level assessment (see item 12).	8, Table S1, Figure 1
Results of individual studies	20	For all outcomes considered (benefits or harms), present, for each study: (a) simple summary data for each intervention group and (b) effect estimates and confidence intervals, ideally with a forest plot.	8, Table 2
Synthesis of results	21	Present results of each meta-analysis done, including confidence intervals and measures of consistency.	Table 2, Figures 2-4
Risk of bias across studies	22	Present results of any assessment of risk of bias across studies (see Item 15).	Table S2
Additional analysis	23	Give results of additional analyses, if done (e.g., sensitivity or subgroup analyses and meta-regression [see Item 16]).	8-9, Table S2
**DISCUSSION**			

Summary of evidence	24	Summarise the main findings including the strength of evidence for each main outcome; consider their relevance to key groups (e.g., healthcare providers, users, and policy makers).	9-10
Limitations	25	Discuss limitations at study and outcome level (e.g., risk of bias) and at review-level (e.g., incomplete retrieval of identified research, and reporting bias).	11-12
Conclusions	26	Provide a general interpretation of the results in the context of other evidence, and implications for future research.	9-12
**FUNDING**			
Funding	27	Describe sources of funding for the systematic review and other support (e.g., supply of data) and role of funders in the systematic review.	14
